# Protracted molecular dynamics and secondary structure introspection to identify dual-target inhibitors of Nipah virus exerting approved small molecules repurposing

**DOI:** 10.1038/s41598-024-54281-9

**Published:** 2024-02-14

**Authors:** Siyun Yang, Supratik Kar

**Affiliations:** Chemometrics and Molecular Modeling Laboratory, Department of Chemistry and Physics, Kean University, 1000 Morris Avenue, Union, NJ 07083 USA

**Keywords:** Computational biology and bioinformatics, Drug discovery

## Abstract

Nipah virus (NiV), with its significantly higher mortality rate compared to COVID-19, presents a looming threat as a potential next pandemic, particularly if constant mutations of NiV increase its transmissibility and transmission. Considering the importance of preventing the facilitation of the virus entry into host cells averting the process of assembly forming the viral envelope, and encapsulating the nucleocapsid, it is crucial to take the Nipah attachment glycoprotein-human ephrin-B2 and matrix protein as dual targets. Repurposing approved small molecules in drug development is a strategic choice, as it leverages molecules with known safety profiles, accelerating the path to finding effective treatments against NiV. The approved small molecules from DrugBank were used for repurposing and were subjected to extra precision docking followed by absorption, distribution, metabolism, excretion, and toxicity (ADMET) profiling. The 4 best molecules were selected for 500 ns molecular dynamics (MD) simulation followed by Molecular mechanics with generalized Born and surface area solvation (MM-GBSA). Further, the free energy landscape, the principal component analysis followed by the defined secondary structure of proteins analysis were introspected. The inclusive analysis proposed that Iotrolan (DB09487) and Iodixanol (DB01249) are effective dual inhibitors, while Rutin (DB01698) and Lactitol (DB12942) were found to actively target the matrix protein only.

## Introduction

Nipah virus (NiV) is a significant public health concern due to its severe impact on human health. Originating from bats, this virus has been responsible for outbreaks causing encephalitis and respiratory diseases in humans with death occurring 7–10 days after infection^1–4^. NiV was primarily identified in Malaysia among workers from one abattoir who had contact with pigs in 1999 resulting in encephalitis in 9 of the patients. It is then spreading out among major southeast countries including Bangladesh, India, Singapore, and the Philippines. The most recent outbreak was in Kerala, India, where the virus infected six individuals, resulting in two deaths^5^. Statistical data reported by Yang and Kar illustrated that the mortality rate of NiV in India and Bangladesh is more than 70%^6^. The high mortality rate underlines the pressing necessity to develop viable strategies to counteract the impacts of the NiV. Additionally, in upcoming years there is a possibility of strong mutation of NiV in their reservoir followed by zoonotic transfer with a high R_0_ value generating a possibility of higher transmission rate as a single strand RNA virus^2,7^. The combination of higher transmission and mortality rate of NiV can be a reason for a much deadlier Pandemic compared to COVID-19^8^. Currently, no specific small molecule treatment or vaccine is approved for NiV^9^. The standard care is supportive, focusing on symptom management and hydration^9,10^. Although research is ongoing, with advancements in monoclonal antibodies and vaccine development, these are still in clinical trial phases and not yet available for widespread use^11,12^.

In the absence of approved small molecule treatment, computational drug repurposing presents a promising avenue^13^. This approach leverages existing approved drugs, potentially accelerating the discovery and deployment of effective treatments like Thalidomide for multiple myeloma and Ivermectin for river blindness^14,15^. Small molecule therapeutics play a pivotal role in drug development due to their size, which allows them to interact with biological targets easily^16^. Computer-Aided Drug Design (CADD), a technique that has revolutionized the field of medicinal chemistry, stands at the forefront of this endeavor^17^. By leveraging computational power to analyze biological and chemical data, CADD can quickly identify promising drug candidates, potentially accelerating the transition from laboratory research to clinical trials^18^. Meanwhile, by targeting specific proteins or pathways known to be involved in the virus's lifecycle, CADD could facilitate the identification of drugs that can be repurposed to fight the virus more effectively and promptly^19^. In the previous research on the CADD of the NiV, quinolone derivatives were screened by Niedermeier et al., which showed high potential as a Nipah Virus Fusion protein inhibitor^20^; James et al. studied 22 favipiravir derivatives in 2021 to target the Nipah attachment glycoprotein^21^. Furthermore, Lee conducted a potential efficacy against three different protein targets of NiV^22^. However, there remains a notable gap in research concerning the comprehensive analysis of these drugs' effects on dual or multiple viral targets, which is essential for a more effective therapeutic response. Additionally, there is a lack of extended MD simulations in existing studies, which are crucial for understanding the dynamic interactions between drugs and viral proteins over time. Still, the defined secondary structure of protein analysis can be another critical point that has been neglected over the years for the NiV drug design and discovery.

**Figure 1:**
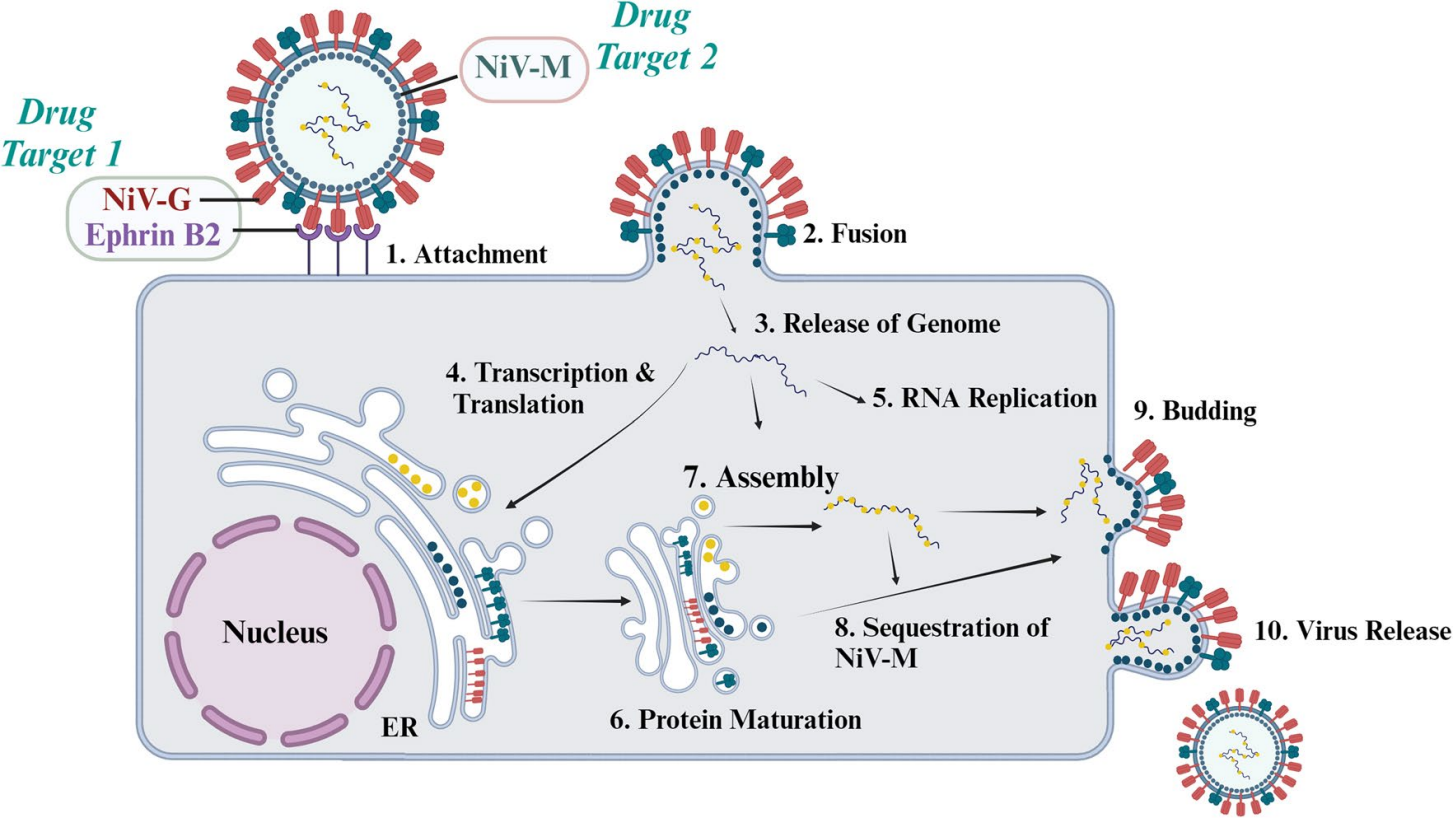
Nipah attachment glycoprotein (NiV-G) (**a**) and Nipah matrix protein (NiV-M) working mechanism (**b**). “Created with BioRender.com”.

Considering the complex nature of NiV, dual-target inhibitors, which act on two proteins, could offer a more effective therapeutic strategy. Targeting multiple stages of the virus lifecycle might enhance efficacy and reduce the likelihood of resistance development. In the present study, we have targeted NiV attachment glycoprotein-human ephrin-B2 (NiV-G) and matrix protein (NiV-M) as dual targets. The major reasons are the following: NiV was found to infect cells through their fusion and attachment glycoprotein by a pH-independent membrane fusion process^23^. Among the process, Ephrin-B2 is identified as the primary tropism of the NiV and the role of glycoprotein in facilitating the virus's entry into host cells through high-affinity protein–protein interactions is essential to treat glycoprotein as a primary target in preventing infections^23–25^. The paramyxovirus matrix (M) is a key target for antivirals responsible for protein-mediating virion assembly and budding from host cell membranes^26^. Simultaneously, as previously identified, the loss or mutation of M severely impairs viral replication, and the significance of matrix protein inhibitor discovery is clear and present^27–29^. The previous experience with Amantadine indicated that matrix protein can be an effective antiviral drug design target^30^. The working mechanism of two selected proteins in the NiV replication cycle was shown in Fig. 1. Our study employs extended molecular dynamics and secondary structure introspection to identify potential dual-target inhibitors. These methods enable a deeper understanding of potential inhibitors' interactions with viral proteins, ensuring more effective and targeted inhibitors for the treatment of NiV.

## Results

### Binding site prediction, receptor grid generation, and docking result

The Sitemap module of Schrodinger was used in the active site identification process^31^. As the protein has no co-crystalized ligand, SiteScore was used for further study. We’ve reported an active site study of NiV attachment glycoprotein in complex with human cell surface receptor ephrinB2 (NiV-G) (PDB ID: 2VSM) in our previous work^32^. The receptor grid generation was conducted at coordinates X: 32.57, Y: 60.01, and Z: 35.22, utilizing a midpoint box with a dimension of 13 Å for the ligand diameter and 20 Å for dock ligands length. For Crystal structure of NiV matrix protein (PDB ID: 7SKT) is predicted with a SiteScore of 1.026 including the amino acids of Chain A: GLY122, SER123, THR124, GLU125, ASN152, ALA153, VAL154, LYS155, MET188, ILE189, PRO190, ARG191, LEU194, LEU237, HIP238, CYS299, PHE300, SER301, ASP304, ILE305, PRO307; Chain B: GLY122, SER123, THR124, GLU125, ASN152, ALA153, VAL154, LYS155, MET188, ILE189, PRO190, ARG191, LEU194, LEU237, HIP238, LEU298, CYS299, PHE300, SER301, ASP304, ILE305, PRO307. The same basic receptor generation setting was used at the center of X: 7.3, Y: 16.46, and Z: 31.83. Both active sites are shown in Fig. S1. We performed extra precision docking experiments involving 6918 prepared ligands conformation with two preselected proteins. Our evaluation criteria were established based on protein docking scores with specific cut-off values, as follows: for 2VSM, a negative score greater than − 10 kcal/mol, and for 7KST, a negative score exceeding − 8 kcal/mol. Following the implementation of these cut-off values in our analysis, we identified eight distinct ligands that exhibited high performance across both proteins. These selected ligands (structure shown in Fig. S2), which are delineated in Table 1, were used advanced in further ADMET studies.

**Table 1:** Docking results of top 8 identified ligands from DrugBank.

PDB ID	DrugBank ID	Docking score	XP Gscore	Glide energy	Glide ligand efficiency
2VSM	DB00290	− 10.492	− 11.702	− 95.202	− 0.109
7SKT	DB00290	− 8.756	− 9.966	− 92.738	− 0.091
2VSM	DB00644	− 10.103	− 10.645	− 82.344	− 0.119
7SKT	DB00644	− 9.074	− 9.615	− 98.007	− 0.107
2VSM	DB01249	− 10.56	− 10.56	− 92.554	− 0.17
7SKT	DB01249	− 8.667	− 8.667	− 67.308	− 0.14
2VSM	DB01698	− 10.054	− 10.089	− 64.001	− 0.234
7SKT	DB01698	− 9.026	− 9.061	− 54.684	− 0.21
2VSM	DB09487	− 15.183	− 15.342	− 77.703	− 0.227
7SKT	DB09487	− 8.774	− 8.932	− 78.171	− 0.131
2VSM	DB11602	− 10.925	− 10.295	− 64.682	− 0.199
7SKT	DB11602	− 9.635	− 9.635	− 61.13	− 0.175
2VSM	DB12942	− 10.858	− 10.858	− 41.776	− 0.472
7SKT	DB12942	− 8.71	− 8.71	− 36.973	− 0.379
2VSM	DB15617	− 12.515	− 12.515	− 59.389	− 0.368
7SKT	DB15617	− 9.286	− 9.286	− 51.448	− 0.273

For 2VSM, we can observe that several key amino acids were recurrently implicated as primary contributors in forming various bonds; the protein–ligand 2D interactions are shown in Fig. S3. Notably, ARG A402 emerged as a critical participant, engaging in numerous interactions, including H-bonds, Pi-cation bonds, and even forming a salt bridge in certain instances. Similarly, amino acids like GLU B97, LYS B95, and ASN B98 were frequently involved, predominantly in H-bond formations, showcasing their repeated participation across different ligand interactions. THR B99 also displayed considerable versatility, being part of H-bond and Pi-cation interactions in various occurrences. Furthermore, amino acids LYS B80 and GLU A501 consistently participated in H-bond interactions, indicating their potential role as pivotal interaction sites. Other recurring amino acids participating in H-bond formations include ARG A242, ASP B149, and LEU A305.

Upon evaluating the protein–ligand interactions concerning the protein 7KST (showed in Fig. S4), we identified a series of amino acids that play a pivotal role in facilitating bond formations with various ligands. The residue ASP A304 consistently stands out as a critical participant, primarily engaging in hydrogen bonding and further contributing to the formation of salt bridges, thereby hinting at its significant role in augmenting the stability of the protein–ligand complexes. Similarly, the ARG B191 residue marks its presence recurrently, predominantly involving itself in hydrogen bond formations while also participating in halogen bond interactions on certain occasions. This recurring involvement underscores its vital role in enhancing ligand binding. Concurrently, ASP B304 consistently appears as a central figure in the interactions, especially in facilitating hydrogen bonds, thereby suggesting its central role in the ligand anchoring process. Furthermore, residues LYS A155 and LYS B155 emerge as notable elements, recurrently engaging in hydrogen and Pi-cation interactions, which delineate their potential importance in stabilizing the protein–ligand assemblies. Additionally, residues ASN A152 and ASN B152 demonstrate a significant propensity to be involved in hydrogen bond formations with various ligands, indicating their potential as pivotal points in establishing potent ligand affinities.

### ADMET profiling

The top 8 approved small molecules were selected according to docking score, binding free energy, and Glide ligand efficiency, which were subsequently evaluated for their ADMET properties. The full table of ADMET profiling is shown in Table S2. Regarding our predicted result, five of our ligands were identified as class 5 and 6, a high value of class indicating less toxicity and a higher value of LD_50_. Taking the Predicted Toxicity Class greater or equal to 5 as a primary filter, five ligands were selected and 3 were excluded. Deep green color cells indicate strong inactive nature of the specific ligand towards toxicity endpoints while deep red indicates strong active. With the number in the cell specifying the confidence of our prediction. We can see that most of our predictions shown in Table 2 are relatively confident results. Considering the confidence value and the predicted toxicity class for each category, the only ligand that needs to be aware of is the DB01698. However, Immunotoxicity refers to the adverse effects on the functioning of the immune system produced by exogenous agents^33^. Such interference with the immune function can manifest as suppression, leading to heightened vulnerability to infections, tumors, or exaggeration, potentially resulting in allergic reactions or autoimmune diseases. Considering all the above factors, we have chosen DB00644, DB01249, DB01698, DB09487, and DB12942 for further Molecular Dynamics study.

**Table 2:**
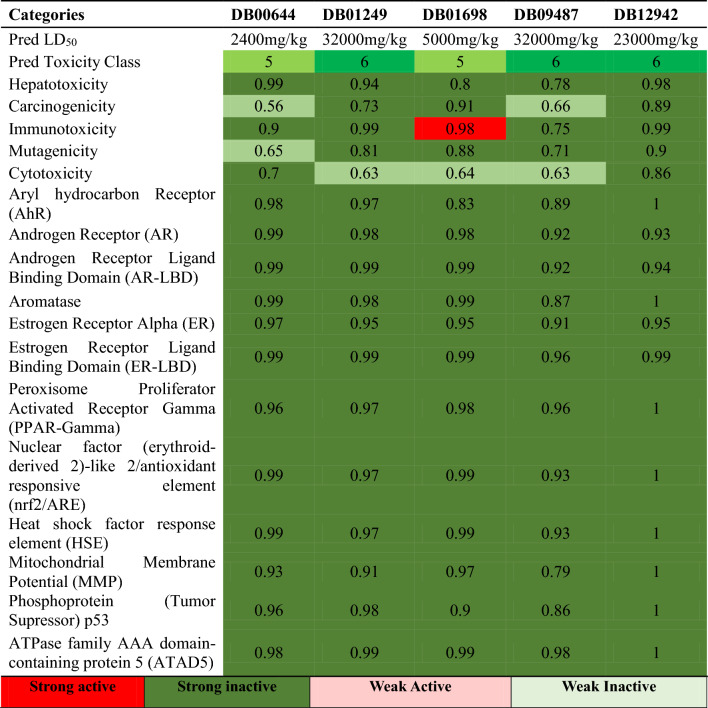
ADMET profiling of top 5 screened ligands from XP-Glide docking study from DrugBank database.

### Molecular dynamics and prime MM-GBSA study

**Figure 2:**
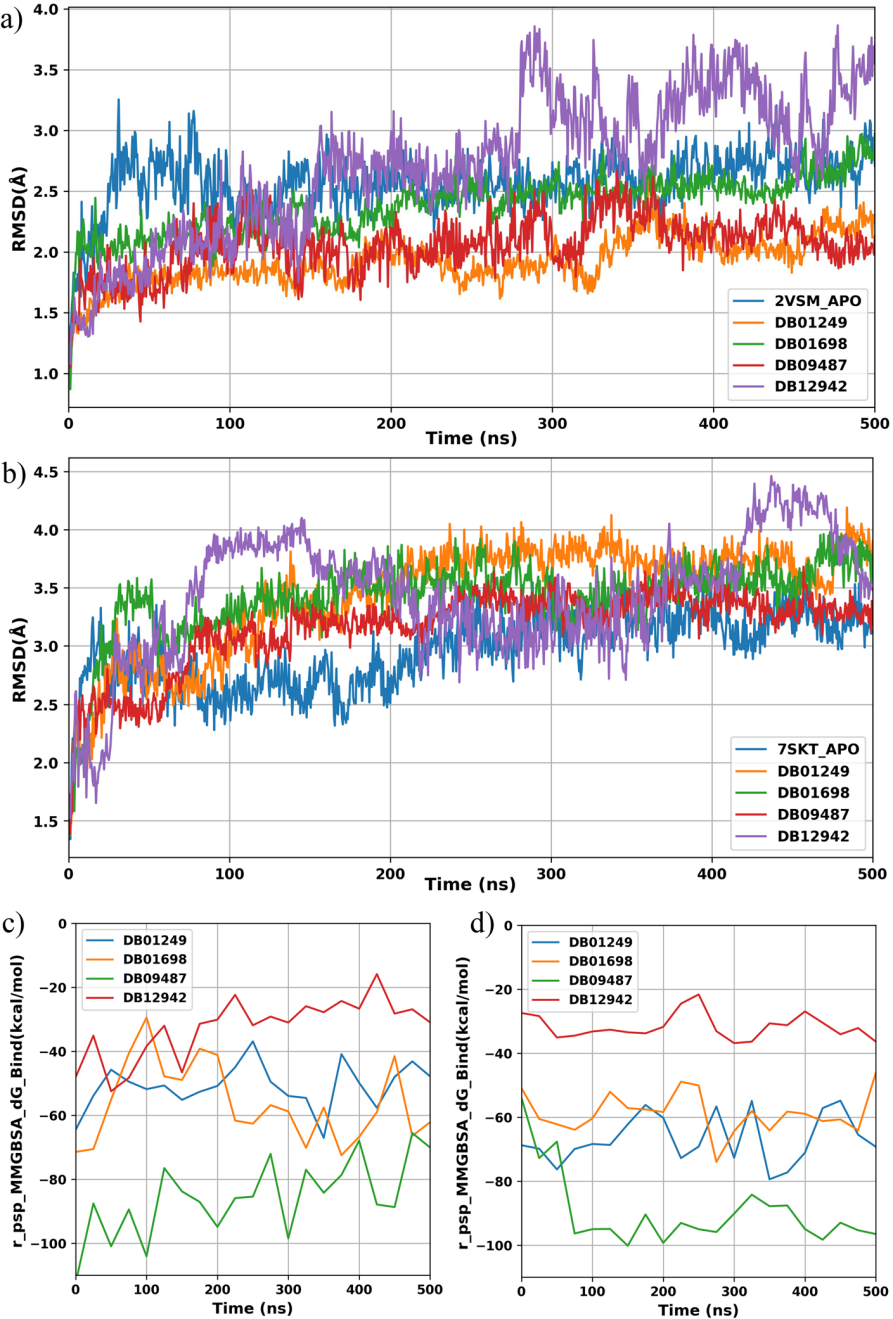
RMSD alignment analysis among apo-protein and ligand-complexes for NiV-G (PDB ID: 2VSM) (**a**) and for NiV-M (PDB ID: 7SKT) (**b**) based on 500 ns MD analysis. MMGBSA_dG_Binding value line chart for 500 ns MD simulation for NiV-G (**c**) and NiV-M (**d**).

The five molecules selected from the ADMET study were used in the MD simulations. All the MD simulation results were performed by the Desmond module of Schrodinger, and obtained trajectories were analyzed using the Simulation Interaction Diagram and were reported in the Supplementary Information Figs. S5–S14. From our result, the RMSD value of DB00644 in the complex with 2VSM protein we observed a large variation between protein and ligand for around 400 ns, indicating a potential ligand off-target from our active site. For this reason, DB00644 was excluded from the further analysis. Figure 2a,b showed protein RMSD value indicating the conformation change of the complex protein compared to the apo-protein. For 2VSM, other than DB012942, all the other protein–ligand complexes tended to stay in stable states with relatively small RMSD value changes. Again, it’s important to mention that although DB012942 deviates from the APO-protein but never more than 1.5 Å during the 500 ns. While, in the case of 7SKT, all protein–ligand complexes never deviated more than 1.5 Å throughout 500 ns. Since all four protein–ligand complexes have stable RMSD values, MM-GBSA binding energy was used for further evaluation.

The Molecular Mechanics energies combined with the Generalized Born and Surface Area continuum solvation (MM-GBSA) line plots (Fig. 2c,d) presented for ligands interacting with protein targets 2VSM and 7SKT provide insights into the binding affinity over time during molecular dynamics simulations. The relatively lower r_psp_MMGBSA_dG_Binding value suggests stronger binding affinity, which is a desirable characteristic for potential inhibitors. For protein 2VSM, ligands DB01249 and DB09487 demonstrate the most consistently low binding free energy throughout the 500 ns simulation, indicating a stable interaction with the target. For DB01249, the binding energy was consistent around – 40 to − 60 kcal/mol, which is more stable and has lower energy than the other ligands. DB9487 showed the lowest binding energy across the time. In the case of protein 7SKT, the trend is similar. DB01249 maintains a consistently strong binding affinity across the simulation period, maintaining binding energy between – 60 to − 80 kcal/mol. At the same time, DB09487 shows some fluctuation yet sustains a notably lowest binding energy compared to the other ligands after 70 ns. This suggests that both DB01249 and DB09487 have favorable interactions with 7SKT. The observed stability and low binding free energy for these ligands across two different protein targets highlight their potential as effective inhibitors, corroborating that DB01249 and DB09487 are the best ligands for both proteins.

**Figure 3:**
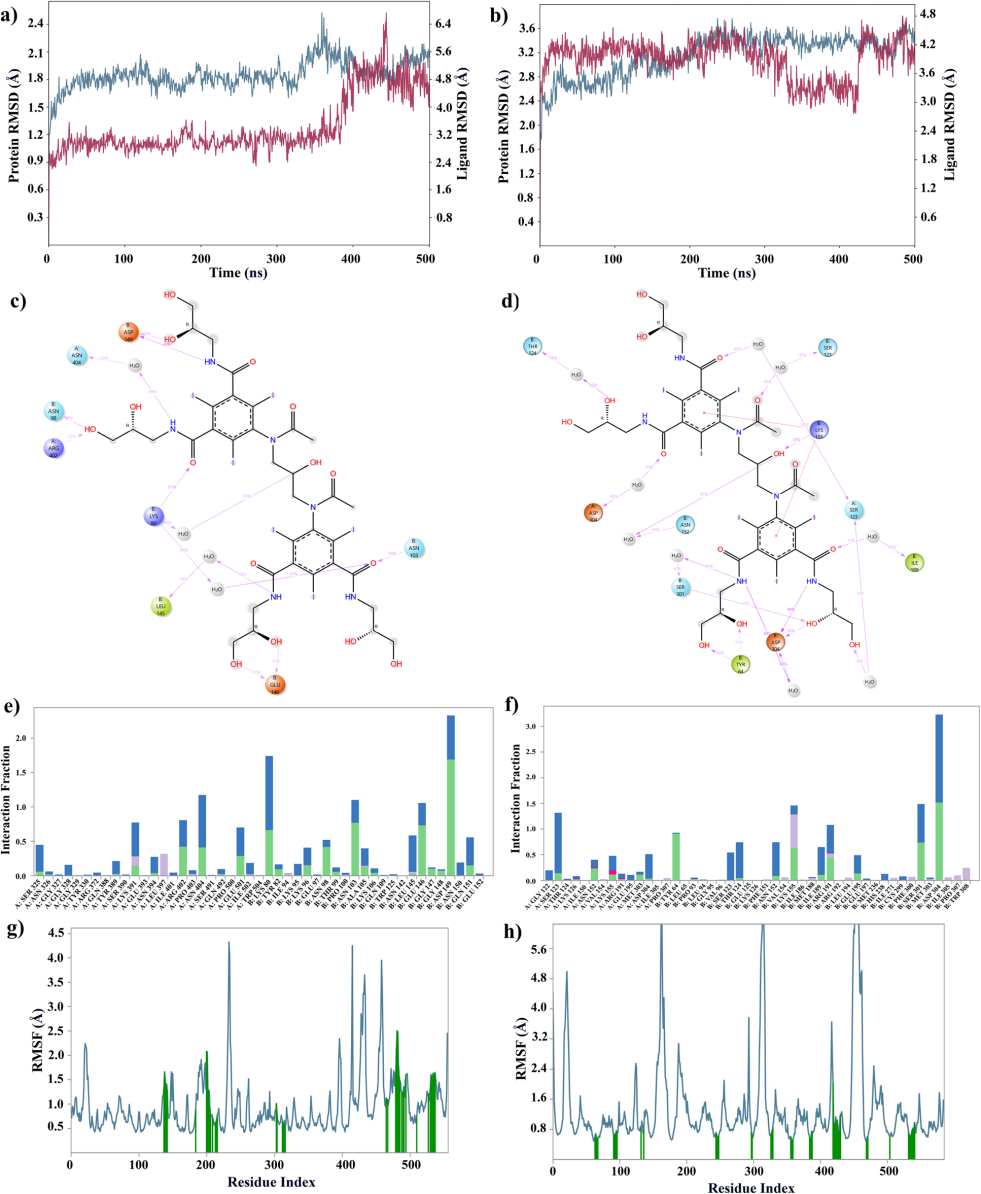
Result of 500 ns MD analysis for DB01249 binding to dual targets. The protein–ligand RMSD plot of DB01249 bound to NiV-G (PDB ID: 2VSM) (**a**) and NiV-M (PDB ID: 7SKT) (**b**). Ligand–protein contact interactions scheme with the protein residues of DB01249 bound to 2VSM (**c**) and 7SKT (**d**) protein. Protein–ligand contacts histogram of the interaction fraction of H-bond (green), hydrophobic bond (Purple), ionic bond (magenta), and water bridges (blue) for 2VSM (**e**) and 7SKT (**f**). RMSF plot of 2VSM (**g**) and 7SKT (**h**) protein–ligand complex.

Detailed analyses regarding DB01249 and DB09487 are shown below. Figure 3a represents the DB01249 ligand in complex with the 2VSM, the RMSD values of the protein (blue line) remain relatively low and stable throughout the 500 ns simulation time, with a slight upward trend but without significant fluctuations. This suggests that the protein structure does not deviate much from the initial conformation, indicating good stability. The ligand RMSD (red line) also appears stable, though it shows more variability than the protein. At the first 400 ns of this indicates that the ligand may have some flexibility in the binding site but overall maintains consistent interaction with the protein. Within the first 400 ns, even though we didn’t observe a high overlap between ligand and protein, the RMSD difference is stably within 1.4 Å. In the last 100 ns, we can see an overlap between protein and ligand. In the Fig. 3b, which shows the DB01249 ligand with the 7SKT, both the protein and ligand RMSD values fluctuate more than in the attachment glycoprotein complex but remain within a relatively narrow range. This indicates a stable interaction with a higher degree of structural flexibility. Our observed RMSD value is within 0.4 Å throughout the 500 ns period. The comparable low and stable RMSD values for the protein and ligand in each complex suggest that DB01249 forms a stable complex with the Nipah virus attachment glycoprotein and the matrix protein. This stability is a positive indicator that DB01249 could potentially inhibit the function of both proteins.

Introspecting Fig. 3c,d, the H-bond and water bridge are dominant in the binding process. In the 2VSM protein–ligand complex, ASP B149 forms an H-bond with the amine group in the ligand structure 64% of the time, while 57% of the time, LYS B80 also has formed an H-bond with the carbonyl group. Meanwhile, we can observe that there are eight amino acids involved in the protein–ligand interaction, which will make the complex tend to be stable. For Fig. 3e,f, we can still observe the dominance of the H-bond and water bridge. However, we have the ionic bond participating in this case. Notably, the ASP B304 forms an H-bond between an amine group, a hydroxyl group, and water during 84%, 55%, and 68% of the time, respectively. Ten different amino acids were involved in this protein–ligand complex.

Root Mean Square Fluctuation (RMSF) plots illustrate the flexibility of each residue in the protein throughout the simulation. Lower values indicate less movement from the average position, suggesting a more rigid, structured part of the protein. Higher values indicate more flexibility. Complementing this, our RMSF plots (Fig. 3g,h) shed light on the dynamic behavior of protein residues upon ligand binding. The RMSF data for both 2VSM and 7SKT complexes suggest that the presence of DB01249 does not lead to significant increases in structural flexibility, which is often associated with destabilization. Rather, the ligand seems to maintain or even enhance the structural integrity of the proteins.

**Figure 4:**
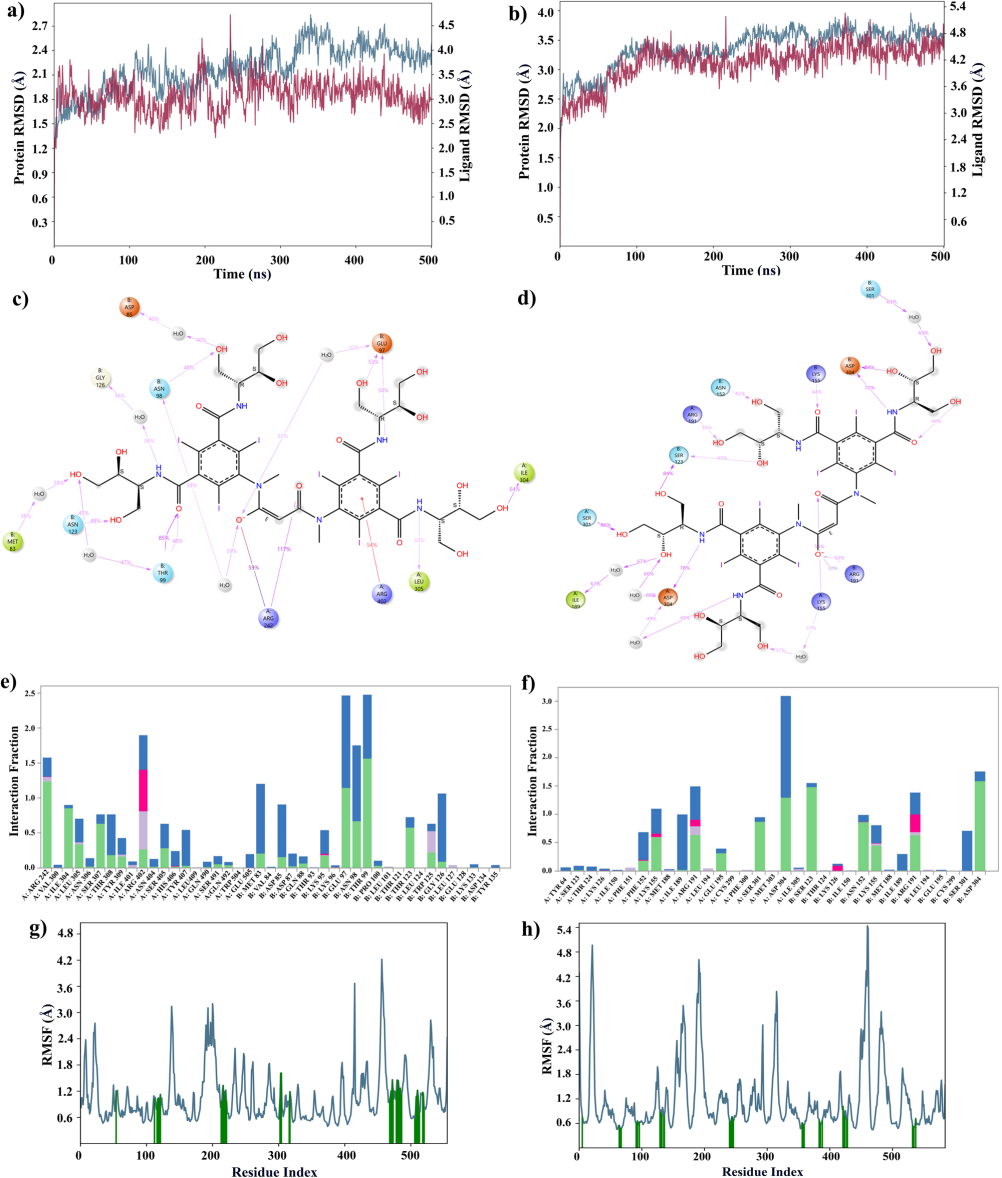
Result of 500 ns MD analysis for DB09487 binding to dual targets. The protein–ligand RMSD plot of DB09487 bound to NiV-G (PDB ID: 2VSM) (**a**) and NiV-M (PDB ID: 7SKT) (**b**). Ligand–protein contact interactions scheme with the protein residues of DB09487 bound to 2VSM (**c**) and 7SKT (**d**) protein. Protein–ligand contacts histogram of the interaction fraction of H-bond (green), hydrophobic bond (Purple), ionic bond (magenta), and water bridges (blue) for 2VSM (**e**) and 7SKT (**f**). RMSF plot of 2VSM (**g**) and 7SKT (**h**) protein–ligand complex.

The RMSD plots (Fig. 4a,b) provided for the MD simulations of the DB09487 ligand with two NiV proteins offer key insights into the dynamic behavior of these complexes over a 500 ns simulation period. In the case of the DB09487-2VSM complex and the DB09487-7SKT complex, the RMSD values for the protein fluctuate around a relatively narrow range, suggesting that the protein maintains a stable conformation throughout the simulation. The ligand RMSD also shows limited fluctuation, indicating that once bound, the ligand remains consistently positioned within the binding site of the attachment glycoprotein. In the RMSD plot, we observed that the difference between both protein and ligand RMSD values are all around 1 Å. The minimal deviation of the ligand RMSD from the protein RMSD suggests a synergistic stability between the ligand and the protein, an indication of a stable complex that is less likely to dissociate under physiological conditions. This result suggests the hypothesis that DB09487 has the potential to act as an inhibitor for both the Nipah virus attachment glycoprotein and matrix protein.

Introspecting Fig. 4c,d, it is interesting that ARG A242 is forming an H-bond with a carboxyl group 117% of the time. A value higher than 100% indicates this residue has multiple interactions of a single type of bond with the same ligand atom. Meanwhile, a soft bridge exists between ARG A242 and the hydroxyl group 59% of the time. The histogram indicated our dominant bonds were H-bond and water bridge, ARG A242, ARG A402, GLU B97, ASN B98, and THR B99 were major protein–ligand interaction involvers (Fig. 4e,f). Analyzing the protein–ligand contacts for 7SKT (Fig. 4f), the ASP amino acid on both chains is pivotal in the protein–ligand interactions. The ASP 304 on chain A forms H-bonds to the amine group and the water molecule, which helps the stabilization of the protein–ligand complex in the solvent system. The ASP 304 on chain B forms the H-bonds with both the amine group and the hydroxyl group. Notably, a soft bridge interaction was also formed between the hydroxyl group and ARG B191 in the case of 7SKT.

RMSF plots (Fig. 4g,h) are indications of the two protein behaviors during the ligand binding process. Considering the overall performance of DB9487 with NiV-G and NiV-M, the relatively low and stable RMSF value can also indicate that this ligand might be contributing to reduced flexibility in these regions, potentially affecting the protein's interaction with other molecules or its role in the viral lifecycle.

### Free energy landscape (FEL) and principal component analysis (PCA) interpretation

**Figure 5:**
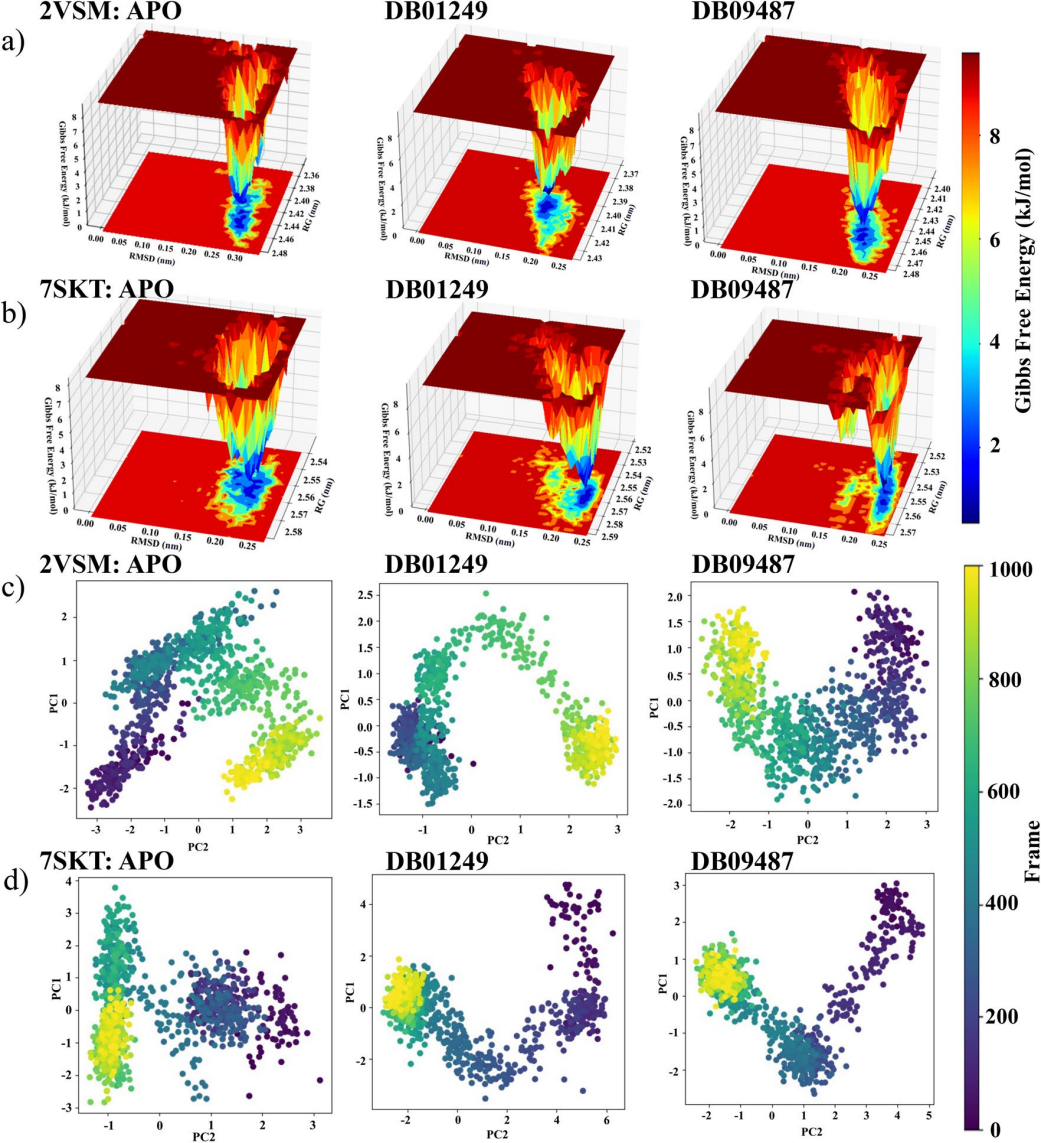
FEL and PCA plot analysis for 2VSM and 7SKT. FEL plots regarding apoprotein, DB01249, and DB09487 bound to NiV-G (PDB ID: 2VSM) (**a**); and NiV-M (PDB ID: 7SKT) (**b**). PCA plots regarding apoprotein, DB01249, and DB09487 bound to NiV-G (PDB ID: 2VSM) (**c**); and NiV-M (PDB ID: 7SKT) (**d**).

FEL and PCA analysis are done on all four selected ligands as shown in Figs. S15–S18. For the free energy landscape, the color gradient represents the frequency or probability of the protein being in a particular conformation, with red areas indicating low probability (high energy) states and blue areas indicating high probability (low energy) states. Considering Fig. 5a, the apo form of 2VSM shows a free energy landscape with a distinct minimum, indicating a stable conformation with a low RMSD value. This suggests that in the absence of a ligand, the protein likely adopts a specific structure or a set of closely related structures. The energy barrier to reach higher RMSD values is significant, indicating that conformational changes away from the reference structure are energetically unfavorable in the absence of a ligand. Our ligand DB01249 appears to stabilize a conformation that is close to the reference structure, as indicated by the deep well at a low RMSD value. The conformational change is minimal, suggesting that this ligand may stabilize the native or a slightly altered conformation of the protein. Simultaneously, the landscape of ligand DB09487 shows a deep but broader well, which might imply that the ligand accommodates a variety of conformations or induces a more flexible binding site.

The APO state of 7SKT shows a relatively broad distribution of conformations with low RMSD values, indicating the protein's native or near-native states have a range of stable conformations. The FEL diagram (Fig. 5b) shows the presence of multiple minima, which could correspond to different stable conformations or functional states of the protein. The complex with DB01249 shows a narrow and deep well, indicating a specific and stable binding conformation. The low RMSD values suggest the ligand-bound state does not deviate much from the protein's reference state. The specificity of the well implies that the ligand may induce or stabilize a particular active or binding conformation, which could be associated with high binding affinity and specificity. The complex with DB09487 shows a deep energy well, like the previous ligands, indicative of a stable complex. However, the well is broader, suggesting a greater range of stable conformations when bound to this ligand. The result indicates that the stability binding of our ligands and 7SKT protein as potential targets.

The color gradient in PCA plots (Fig. 5c,d) indicates the progression of frames from the beginning to the end of the simulation, with the starting frames in purple and ending frames in yellow. Principal Component 1(PC1) is the axis along which the data's variance is maximized^34^. In other words, it represents the direction in which the conformational changes of the protein are the greatest. If frames (individual snapshots from the simulation) are clustered close to PC1, it means that the conformational changes are predominantly along the motion captured by PC1. For a protein–ligand complex, this could mean that most of the motion due to binding occurs along the motion described by PC1. It might represent large-scale movements such as domain movements or hinge bending. Principal Component 2 (PC2) captures the second most significant motion, orthogonal to PC1^34^. It’s independent of PC1 and often captures a different type of motion. Frames that are close to PC2 are experiencing a different set of conformational changes than those captured by PC1, and these can often be more subtle, like side-chain rearrangements or small loop movements.

For the attachment glycoprotein, we can observe that the complex with DB01249 appears more constrained, particularly along PC1, than in its apo form, suggesting that the ligand binding reduces the protein's conformational freedom. The clustering of later frames (yellow) indicates that the protein may preferentially adopt and maintain a specific conformation in the presence of this ligand. The DB09487-bound protein samples a different conformational space compared to the apo form, with a distinct separation along PC2, which indicates a progression from early to late frames, suggesting a possible binding-induced conformational change. For the matrix protein, the apo form of the protein samples a wide range of conformations, indicating high flexibility, which is consistent with the matrix protein's multiple roles in virus assembly and lifecycle. The complex with DB01249 shows a conformational distribution that is significantly different from the apo form, with a more compact cluster along PC1, indicating the ligand appears to stabilize the protein, reducing its conformational variability and potentially indicating a binding-specific conformation. The DB09487 complex displays a broad distribution like the apo form, suggesting that while the ligand does bind, it may not restrict the conformational space as much as DB01249.

### Defined secondary structure protein (DSSP) analysis

**Figure 6:**
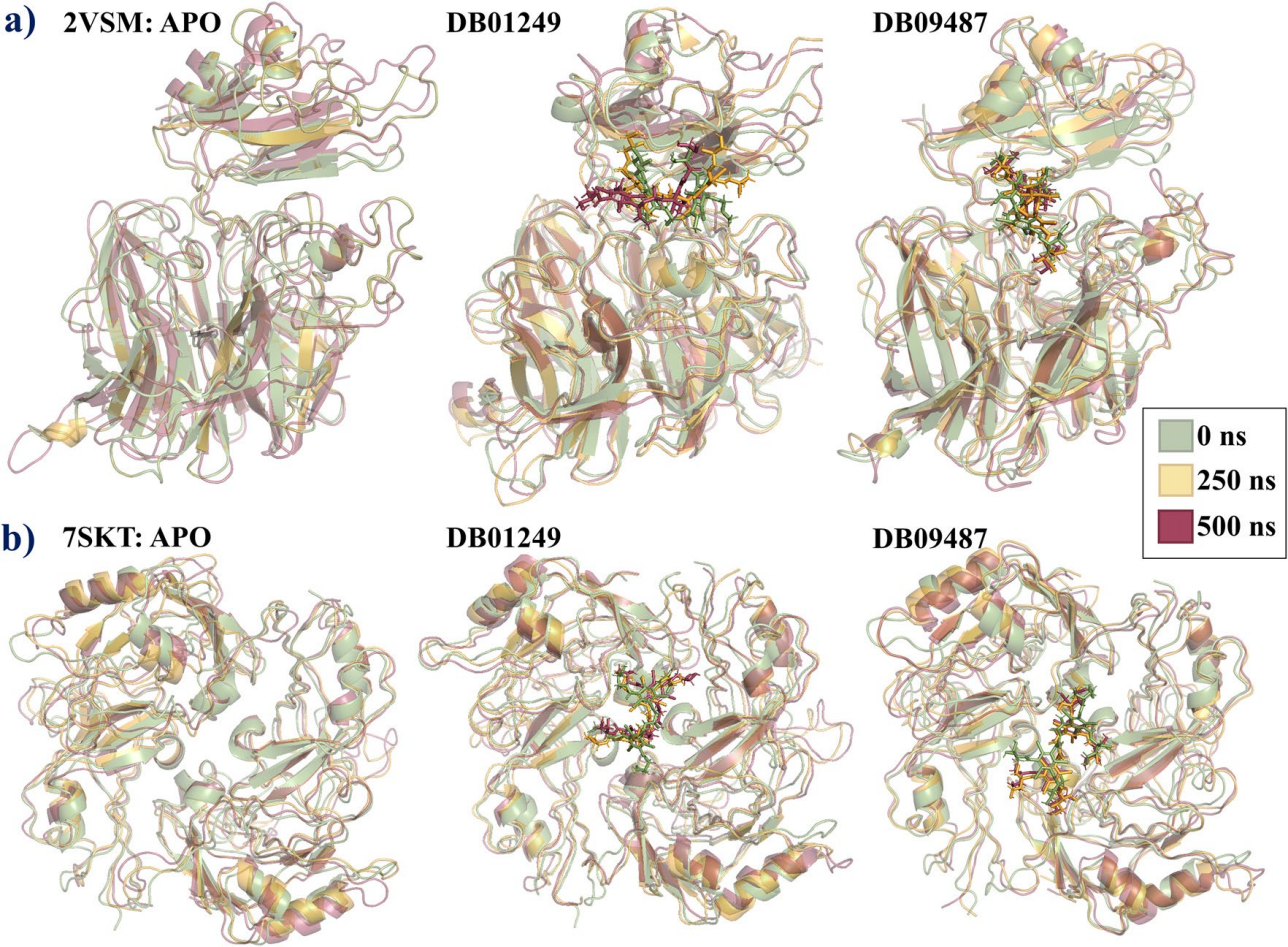
Comparative secondary structure evolution of NiV proteins over time in Apo and Ligand-Bound States. Panel (**a**) represents the structural overlay of the NiV-G (PDB ID: 2VSM) at three simulation time points: 0 ns (green), 250 ns (orange), and 500 ns (red) in its apo form and when bound to ligands DB01249 and DB09487. Panel (**b**) illustrates the corresponding overlays for the NiV-M (PDB ID: 7SKT).

Figure 6 represents the structural changes of two different proteins from the NiV, visualized at different time points (0 ns, 250 ns, and 500 ns) during molecular dynamics (MD) simulations. Over the 500 ns simulation time, the apo form of the attachment glycoprotein maintains its overall secondary structure, as indicated by the strong overlap between the moss green, gold, and maroon colors. This suggests the structural stability of the protein in the absence of a ligand. The binding of DB01249 seems to cause some conformational changes, as evidenced by the divergence of colors, especially noticeable if certain regions shift from moss green to maroon without much gold overlap. This suggests that the ligand induces structural changes that progress over time. Like DB01249, the DB09487 ligand appears to induce changes in the protein structure, with some regions showing a clear progression from moss green through gold to maroon, indicating a gradual change in secondary structural elements. The DSSP analysis for all four ligands can be found in Figs. S19 and S20.

The matrix protein in its apo form also exhibits structural stability, with the colors largely overlapping. This indicates that the protein's secondary structure does not significantly change over the 500 ns timeframe in the absence of a ligand. Ligand DB01249's binding to the matrix protein stabilizes certain regions, as suggested by areas where the overlap is particularly strong. Conversely, regions with less overlap could represent areas where the ligand binding influences the flexibility or induces structural rearrangements. With DB09487, there appears to be a greater degree of structural variation over time compared to the apo form, suggesting that this ligand may have a more pronounced effect on the protein's dynamics.

## Discussion

Multistep virtual screening introspection (Scheme shown in Fig. S21) offered two lead-approved small molecules for NiV dual targets: Iotrolan (DB09487) and Iodixanol (DB01249), possessing high potential for further experimental efforts. Deliberating the other two molecules, Rutin (DB01698) and Lactitol (DB12942), they still showed high aptitude to become effective inhibitors of NiV-M. Our DB01249 complex with the 2VSM protein has a docking score of − 10.56 kcal/mol, while its complex with the 7SKT protein shows a score of − 8.667 kcal/mol. In contrast, the DB09408 complex with the 2VSM protein exhibits a docking score of − 15.183 kcal/mol, and with the 7SKT protein, the score is − 8.774 kcal/mol. Furthermore, the ADMET results revealed predicted low oral LD_50_ toxicity levels, with toxicity grades of 6 for both molecules. The MD study demonstrated that DB009487 exhibited extremely stable overlapping of the ligand and both proteins throughout 500 ns, along with multifaced interactions with amino acids. Similarly, DB01249 also demonstrated good stability throughout the 500 ns of simulations with an RMSD within the fluctuation of 0.5 Å, exhibiting potential interactions of 7SKT protein. Meanwhile, the RMSD between DB01249 and 2VSM is stable within 1.4 Å, indicating a stable protein–ligand complex. Prime MM-GBSA study also confirms their strong free binding energy to validate the reliability of other approaches.

If we compare the structural similarities of four molecules, each has at least one sugar-like substructure. Sugar-like structures can have significant antiviral properties due to glycosylation which involves the addition of sugar molecules to proteins or lipids. Therefore, these molecules can act as glycomimetic which mimic the structure of natural sugars and can interfere with the viral glycosylation process of NiV that may prevent its attachment to host cells or inhibit other steps in the viral life cycle^35,36^. On the contrary, if we introspect two dual-inhibitors (DB01249 and DB0948) only, we can observe that both have nucleobase-like structures and amide linkages, indicating possible peptide-like fragments. DB01698 and DB12942, in contrast, do not contain nitrogen atoms in their structures, precluding the potential for nucleobase-like structures and peptide-like fragments. As nucleobases are the building blocks of nucleic acids, therefore nucleobase-analogs like DB01249 and DB0948 may interfere with the replication of NiV by mimicking the structure of natural nucleotides^37^. While the inclusion of an amide linkage in those molecule structures may enhance their binding and activity against NiV^38^. Considering recent research on the drug repurpose of SARS-CoV-2, our results of DB09487 and DB01249 showed a high overlapped performance on the spike glycoprotein, which indicates the potential of the drug to be a dual virus multiple protein target too^39–41^.

## Methods

### Protein preparation

The protein structure of NiV attachment protein and matrix protein was obtained from the Protein Data Bank (PDB). Currently, a total of 7 NiV-G structures is available in the PDB database. Among these 7, 2VSM has the best X-ray crystallographic resolution (1.80 Å) and the structure is complete without any missing residues^25^. Meanwhile, considering the major receptor role of human ephrin-B2, 2VSM was selected as our potential target. On the other hand, only 2 NiV-M X-ray crystallographic protein structures are available in PDB i.e. 7SKT and 7SKU. Considering the better resolution (2.05 Å) and less interference, 7SKT was selected in our study^27^. All the protein structures were imported into Schrödinger 2023-1 as PDB files^42^. The proteins underwent preparation utilizing the integrated Protein Preparation Workflow tool, which executed tasks of hydrogen addition, disulfide bond allocation, the removal of extraneous water molecules, and charge adjustments. Additionally, missing loops were filled in and termini capped processed with the Prime module, and heterogenous states were generated through Epik. It is essential to note that all water molecules were eliminated prior to initiating the protein preparation phase. To reduce any structural uncertainties, PROPKA was utilized at a pH setting of 7.0 for the optimization of hydrogen bonds, a step critical in rectifying potentially incorrect protonation state assignments within the protein structure, which might otherwise contribute to hydrogen bond network inaccuracies and inducing structural ambiguities. During this phase, the Optimized Potentials for Liquid Simulations 2005 (OPLS_2005) force field was employed, representing a scientifically grounded parameter set devised to accurately forecast atomic behaviors within molecular structures which aimed to minimize potential steric conflicts and refine the protein's overall geometric structure, all while preserving its inherent fold and structural topology. This minimization process persisted until the average root-mean-square deviation (RMSD) for non-hydrogen atoms reached a predefined limit of 0.30 Å. This threshold was established as a controlling parameter, ensuring the maintenance of the protein's macroscopic folding without any significant alterations.

### Active site prediction

To pinpoint the active site of the target protein, we employed the SiteMap tool^31^. This tool leverages a cutting-edge search algorithm to identify potential binding sites present on proteins adeptly. It assesses each site based on various parameters, including its dimensions, volume, amino acid exposure, enclosure, contact, hydrophobic and hydrophilic properties, and evaluating the donor-to-acceptor ratios. At least 15 site points per reported site were generated while 5 site-point groupings were finally generated using the more restrictive definition of hydrophobicity and standard grid. Site maps at 4 Å nearest site point were cropped. The active sites of 7KST were determined by site score and docking results.

### Ligand preparation

We have utilizes the US FDA approved drug as potential ligands which were obtained from the DrugBank Version 5.1.10 Released on 2023-01-04 with drug group called ‘Approved’^43^. The selected file contains a total of 2588 approved drugs whose structures were downloaded and sent through the ligand preparation process. The LigPrep module was utilized for the processing of these ligands, with the maximal ligand dimension configured to encompass 500 atoms, operating under the OPLS_2005 force field^44,45^. Utilizing the Epik ionization tool, the ionization state was established with a target pH range of 7.0 ± 2.0. The procedure facilitated the generation of various protonation and ionization states, stereochemistry, and ring configurations. For those with unassigned chirality, up to 32 stereoisomers per ligand were synthesized. This process culminated in the creation of 6918 potential conformations of ligands, which are slated for subsequent docking analyses.

### Docking

Schrödinger’s Glide software was employed to execute flexible ligand docking utilizing the extra precision (XP) methodology^46,47^. Additionally, penalties determined by the Epik state were incorporated into the docking score assessment. The final scoring leveraged energy-reduced conformations, presenting the outcomes in terms of glide ligand efficiency and the respective docking scores.

### Absorption, distribution, metabolism, excretion, and toxicity (ADMET) prediction

To ensure the efficacy of a new drug candidate, it is crucial to maintain an equilibrium between its drug-likeness attributes and ADMET profiling. Consequently, early-stage predictions regarding the drug-likeness and ADMET properties of potential drug compounds can serve as a preventative measure against costly failures in the later stages of the drug discovery journey^48,49^. The 8 ligands with the best performance overall in the docking study were processed further in the ADMET study. ProTox-II was employed in the ADMET prediction^50^. Due to the conformation shift in the LigPrep procedure, all the structures were built manually in ChemDraw and saved as MOL files. The contents of the MOL files were pasted into ProTox-II for prediction. A total of 19 endpoints were predicted including Predicted LD50, Predicted toxicity class, Average similarity, Prediction accuracy, Hepatotoxicity, Carcinogenicity, Immunotoxicity, Mutagenicity, Cytotoxicity, Aryl hydrocarbon Receptor (AhR), Androgen Receptor (AR), Androgen Receptor Ligand Binding Domain (AR-LBD), Aromatase, Estrogen Receptor Alpha (ER), Estrogen Receptor Ligand Binding Domain (ER-LBD), Peroxisome Proliferator Activated Receptor Gamma (PPAR-Gamma), Nuclear factor (erythroid-derived 2)-like 2/antioxidant responsive element (nrf2/ARE), Heat shock factor response element (HSE), Mitochondrial Membrane Potential (MMP), Phosphoprotein (Tumor Supressor) p53, ATPase family AAA domain-containing protein 5 (ATAD5). Based on the ADMET profiling, 4 molecules were selected for further Molecular Dynamics (MD) simulation.

### Molecular dynamics (MD), free energy landscape (FEL), principal component analysis (PCA) and defined secondary structure protein (DSSP) analysis

The top four chosen ligands were subjected to low-temperature Brownian motion molecular dynamics simulation procedures executed within the Desmond module^51^. These simulations were facilitated on a Linux PC equipped with Nvidia CUDA GPU acceleration, utilizing the 2023 commercial version of Desmond^51^. System systems were prepared for subsequent computations using the System Builder tool integrated within Desmond. The proteins, housed within an orthorhombic enclosure featuring a 10-buffer zone, were resolved utilizing the SPC water model^52^. Leveraging the OPLS_2005 force field, all MD production runs transpired within the NPT ensemble, spanning a simulation duration of 500 ns with system minimization by default method implemented in Desmond. Following this, the C-alpha RMSD trajectory graphs and other illustrative data were generated using the Simulation Interactions Diagram software incorporated in Desmond, facilitating a detailed examination of the trajectories. PyMOL and its plugin Geo-Measures were used for MD trajectory FEL, PCA, and defined secondary structure of proteins analysis^53^. Regarding the DSSP Analysis, the MD trajectory file is loaded to Maestro Schrodinger and the frames of 0 ns, 250 ns, and 500 ns were exported separately with solvent removed. All the structures are aligned in respect to the 0 ns structure.

### MM-GBSA

The Prime MM-GBSA approach calculates the energy of optimized unbound receptors, free ligands, and the receptor-ligand complex. MM-GBSA merges molecular mechanics and solvation models to assess the stability and affinity of the studied receptor-ligand complexes which is extremely in studying protein–ligand interactions. It uncovers the binding free energy by incorporating molecular mechanics' enthalpic contributions and solvation effects from Generalized Born models and Surface Area calculations. Furthermore, it gauges the ligand strain energy by positioning the ligand within a solution independently generated by the VSGB 2.0 suite. The free energy calculation was conducted using a step size of 50 frames. The MM-GBSA_dG_Bind energy was calculated by Complex–Receptor–Ligand^54^.

### Supplementary Information

Supplementary Information.

## Data Availability

Most of the data are already available in the manuscript and Supplementary Information. Any additional data will be made available from the corresponding author on request.
