# Supplementary material for: Protracted molecular dynamics and secondary structure introspection to identify dual-target inhibitors of Nipah virus exerting approved small molecules repurposing
Source: Sci Rep. 2024 Feb 14;14:3696. doi: 10.1038/s41598-024-54281-9 (PMC10866979; doi:10.1038/s41598-024-54281-9)
Supplement: Supplementary file 1 — Supplementary Information. [file 41598_2024_54281_MOESM1_ESM.docx]

**Supporting Information**

**Protracted Molecular Dynamics and Secondary Structure Introspection to Identify Dual-Target Inhibitors of Nipah Virus Exerting Approved Small Molecules Repurposing**

Siyun Yang, Supratik Kar^*^

Chemometrics and Molecular Modeling Laboratory,

Department of Chemistry,

Kean University,

1000 Morris Avenue, Union, NJ 07083, USA

*Corresponding author:

Dr. Supratik Kar ([skar@kean.edu](mailto:skar@kean.edu)); Department of Chemistry, Kean University,

1000 Morris Avenue, Union, NJ 07083, USA. Phone: +1 908-737-3683


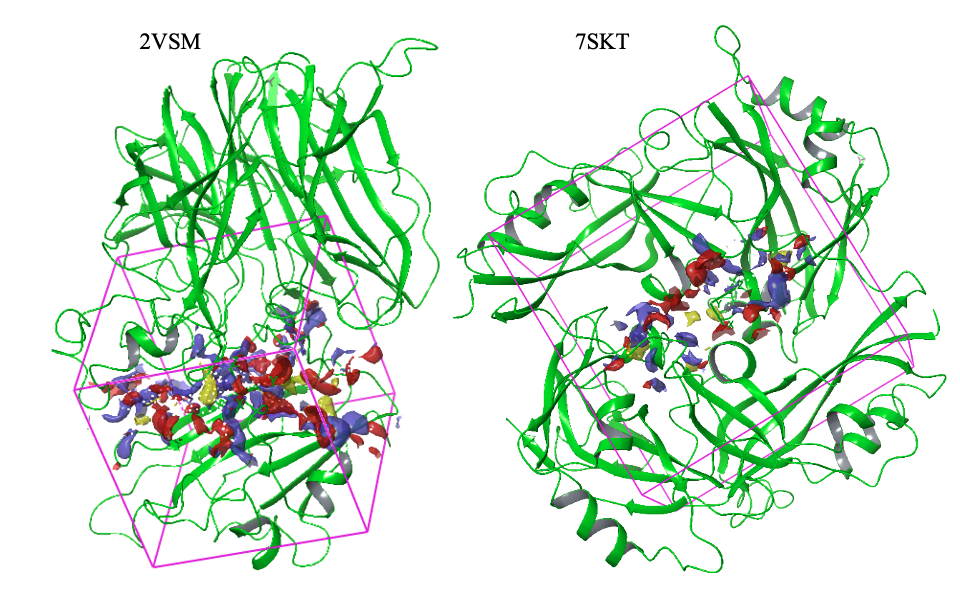


**Figure S1**. Identified active site (pocket) for 2VSM and 7SKT.


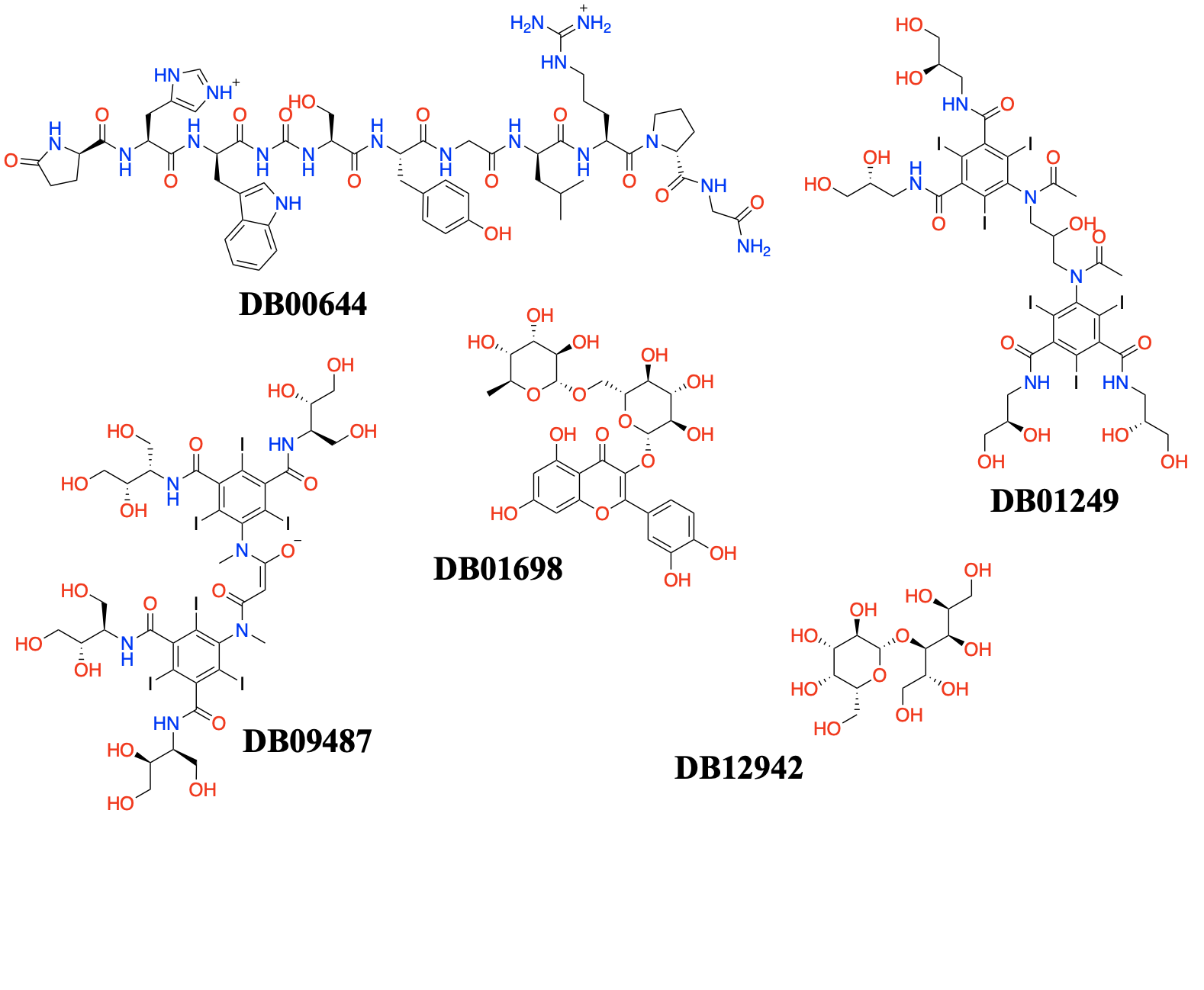


**Figure S2**. 2D structures of top 5 ligands selected from docking and ADMET study.


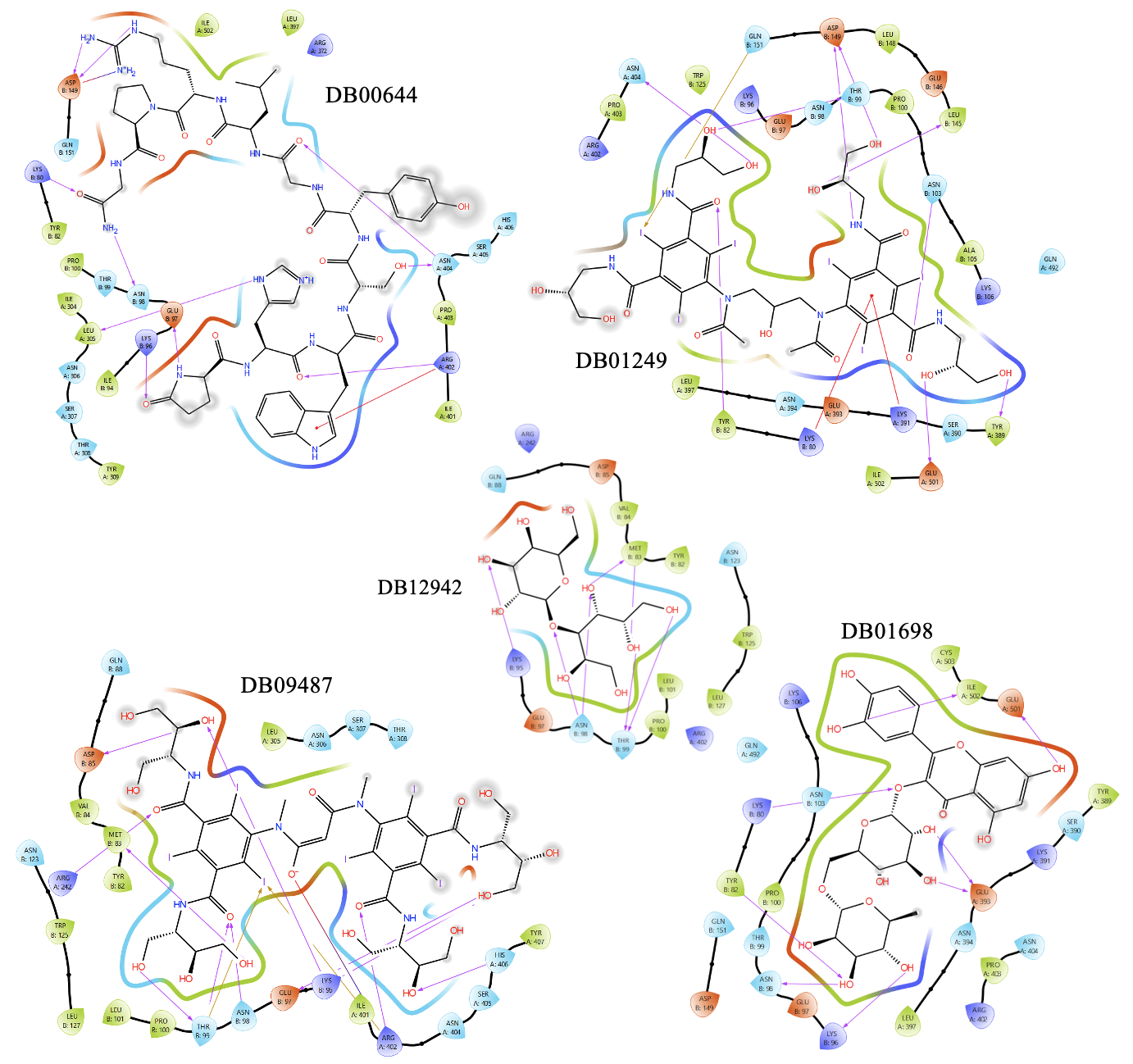


**Figure S3**. 2D Interactions of docking for top 5 ligands with 2VSM protein


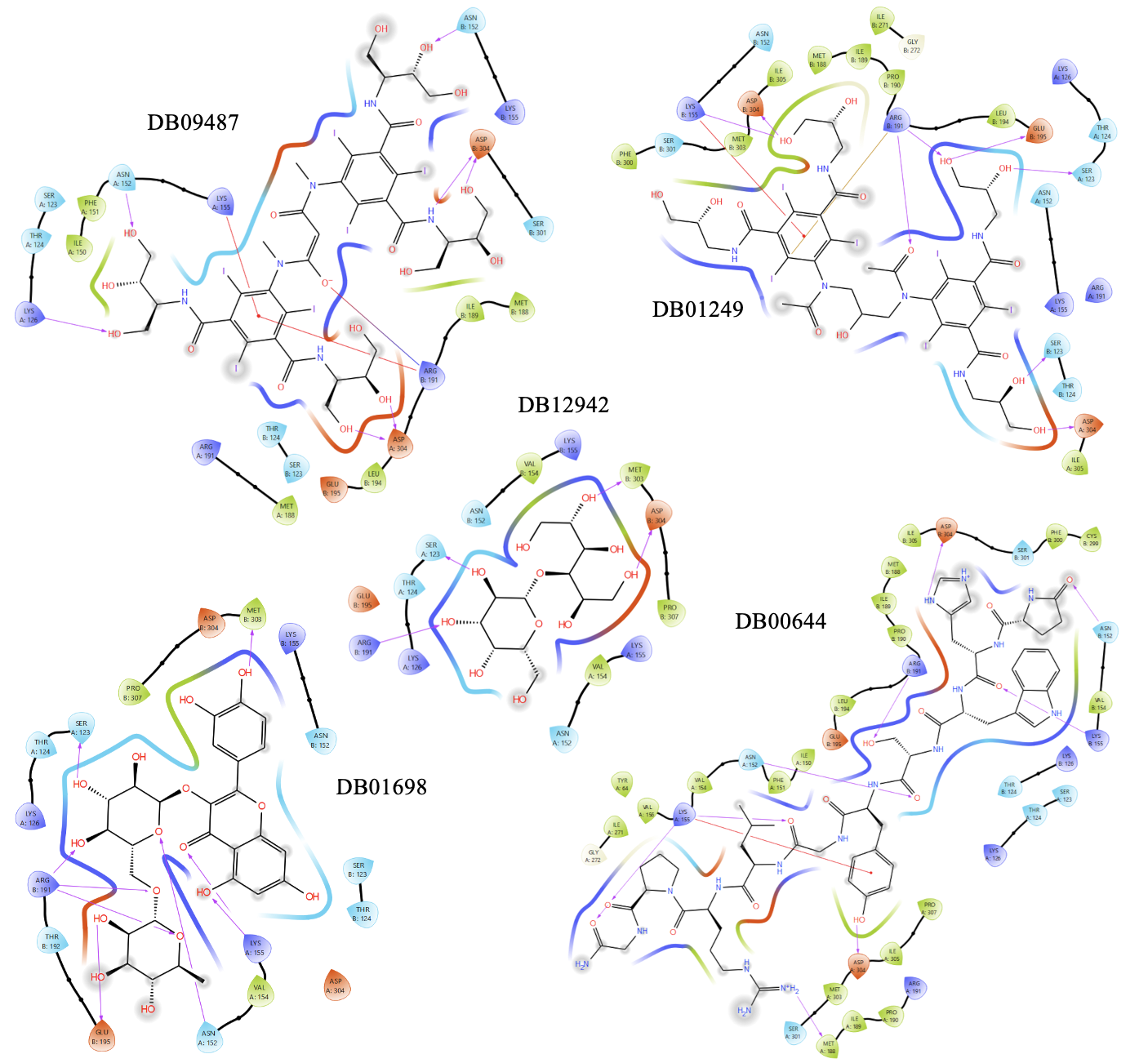


**Figure S4**. 2D Interactions of docking for top 5 ligands with 7SKT protein

**Table S1**. Docking result of top 8 molecules with corresponding docking score, XP Gscore, Glide energy, ligand efficiency, and dual protein-ligand interactions.

| **PDB ID** | **DrugBank ID** | **Docking Score (kcal/mol)** | **XP Gscore** | **Glide Energy** | **Glide Ligand Efficiency** | **Interactions with amino acids** |
| --- | --- | --- | --- | --- | --- | --- |
| 2VSM | DB00290 | -10.492 | -11.702 | -95.202 | -0.109 | ARG A 402: H-bond; LEU A305: H-bond; ARG A 242: H-bond; VAL B84: H-bond; LYS B95: H-bond; GLU B97: H-bond |
| 2VSM | DB00644 | -10.103 | -10.645 | -82.344 | -0.119 | ASN A404: H-bond; ARG A402: H-bond, Pi-cation; LEU A305: H-bond; ASP B149: H-bond; GLU B97: H-bond; LYS B96: H-bond; ASN B98: H-bond; LYS B80: H-bond |
| 2VSM | DB01249 | -10.56 | -10.560 | -92.554 | -0.170 | ASP B149: H-bond; LEU B145: H-bond; ASN B103: H-bond; GLU A501: H-bond; TYR A389: H-bond; LYS A391: Pi-cation; LYS B80: Pi-cation; TYR B82: H-bond; ASN A404: H-bond; THR B99: H-bond; GLN B151: Halogen bond |
| 2VSM | DB01698 | -10.054 | -10.089 | -64.001 | -0.234 | LYS B96: H-bond; TYR B82: H-bond; ASN B98: H-bond; LYS B80: H-bond; GLU A501: H-bond; ILE A502: H-bond; GLU A393: H-bond |
| 2VSM | DB09487 | -15.183 | -15.342 | -77.703 | -0.227 | ASP B85: H-bond; ARG A242: H-bond; MET B83: H-bond; THR B99: H-bond, Pi-cation; ASN B98: H-bond; ARG A402: H-bond, Pi-cation, Salt bridge; LYS B95: H-bond; HIS A406: H-bond |
| 2VSM | DB11602 | -10.925 | -10.295 | -64.682 | -0.199 | ASP B149: H-bond; GLU A501: H-bond; ASN B103: H-bond; PRO B100: H-bond; LYS B80: H-bond; LYS B96: H-bond; GLU A393: H-bond |
| 2VSM | DB12942 | -10.858 | -10.858 | -41.776 | -0.472 | LYS B95: H-bond; MET B83: H-bond; ASN B98: H-bond; THR B99: H-bond; |
| 2VSM | DB15617 | -12.515 | -12.515 | -59.389 | -0.368 | GLN B88: H-bond; LYS B95: H-bond; ASP B85: H-bond; ASN B98: H-bond; MET B83: H-bond; THR B99: H-bond; GLN A490: H-bond; GLU A505: H-bond; ARG A402: H-bond; ARG A242: H-bond; |
| 7SKT | DB00290 | -8.756 | -9.966 | -92.738 | -0.091 | ASP A304: H-bond, Salt bridge; ARG A191: H-bond; ILE A189: H-bond; ARG B191: H-bond; LYS A126: H-bond; ASP B304: H-bond |
| 7SKT | DB00644 | -9.074 | -9.615 | -98.007 | -0.107 | ASN B152: H-bond; ASP B304: H-bond; LYS B155: H-bond; ARG B191: H-bond; ASN A152: H-bond; LYS A155: H-bond, Pi-cation; MET A188: H-bond; ASP A304: H-bond |
| 7SKT | DB01249 | -8.667 | -8.667 | -67.308 | -0.140 | LYS B155: H-bond, Pi-cation; ASP B304: H-bond; ASP A304: H-bond; SER B123: H-bond; ARG B191: H-bond, Halogen bond; GLU B195: H-bond; SER A123: H-bond |
| 7SKT | DB01698 | -9.026 | -9.061 | -54.684 | -0.210 | LYS A155: H-bond; ASN A152: H-bond; GLU B195: H-bond; ARG B191: H-bond; SER A123: H-bond; MET B303: H-bond |
| 7SKT | DB09487 | -8.774 | -8.932 | -78.171 | -0.131 | ASN B152: H-bond; ASP A304: H-bond; LYS A155: Pi-cation; ASN A152: H-bond; LYS A126: H-bond; ARG B191: Pi-cation; ASP B304: H-bond |
| 7SKT | DB11602 | -9.635 | -9.635 | -61.13 | -0.175 | THR A124: H-bond; ASN B152: H-bond; LYS B155: H-bond; ASP B304: H-bond; |
| 7SKT | DB12942 | -8.710 | -8.710 | -36.973 | -0.379 | MET B303: H-bond; ASP B304: H-bond; SER A123: H-bond; ARG B191: H-bond; |
| 7SKT | DB15617 | -9.286 | -9.286 | -51.448 | -0.273 | ASN B152: H-bond; ASN A152: H-bond; LYS B155: H-bond; THR A124: H-bond; SAP B304: H-bond; SER B301: H-bond |

**Table S2**. ADMET profiling of top 8 molecules extracted from the docking outcome.

| **Categories** | **DB00290** | **DB00644** | **DB01249** | **DB01698** | **DB09487** | **DB11602** | **DB12942** | **DB15617** |
| --- | --- | --- | --- | --- | --- | --- | --- | --- |
| Pred LD_50_ | 800mg/kg | 2400mg/kg | 32000mg/kg | 5000mg/kg | 32000mg/kg | 51mg/kg | 23000mg/kg | 51mg/kg |
| Pred Toxicity Class | 4 | 5 | 6 | 5 | 6 | 3 | 6 | 3 |
| Hepatotoxicity | 0.59 | 0.99 | 0.94 | 0.8 | 0.78 | 0.88 | 0.98 | 0.95 |
| Carcinogenicity | 0.53 | 0.56 | 0.73 | 0.91 | 0.66 | 0.71 | 0.89 | 0.92 |
| Immunotoxicity | 0.99 | 0.9 | 0.99 | 0.98 | 0.75 | 0.97 | 0.99 | 0.98 |
| Mutagenicity | 0.87 | 0.65 | 0.81 | 0.88 | 0.71 | 0.74 | 0.9 | 0.9 |
| Cytotoxicity | 0.6 | 0.7 | 0.63 | 0.64 | 0.63 | 0.8 | 0.86 | 0.76 |
| Aryl hydrocarbon Receptor (AhR) | 0.91 | 0.98 | 0.97 | 0.83 | 0.89 | 0.96 | 1 | 1 |
| Androgen Receptor (AR) | 0.9 | 0.99 | 0.98 | 0.98 | 0.92 | 0.91 | 0.93 | 0.63 |
| Androgen Receptor Ligand Binding Domain (AR-LBD) | 0.97 | 0.99 | 0.99 | 0.99 | 0.92 | 0.9 | 0.94 | 0.62 |
| Aromatase | 0.93 | 0.99 | 0.98 | 0.99 | 0.87 | 0.87 | 1 | 0.99 |
| Estrogen Receptor Alpha (ER) | 0.9 | 0.97 | 0.95 | 0.95 | 0.91 | 0.77 | 0.95 | 0.61 |
| Estrogen Receptor Ligand Binding Domain (ER-LBD) | 0.97 | 0.99 | 0.99 | 0.99 | 0.96 | 0.93 | 0.99 | 0.99 |
| Peroxisome Proliferator Activated Receptor Gamma (PPAR-Gamma) | 0.86 | 0.96 | 0.97 | 0.98 | 0.96 | 0.96 | 1 | 0.99 |
| Nuclear factor (erythroid-derived 2)-like 2/antioxidant responsive element (nrf2/ARE) | 0.95 | 0.99 | 0.97 | 0.99 | 0.93 | 0.97 | 1 | 0.99 |
| Heat shock factor response element (HSE) | 0.95 | 0.99 | 0.97 | 0.99 | 0.93 | 0.97 | 1 | 0.99 |
| Mitochondrial Membrane Potential (MMP) | 0.81 | 0.93 | 0.91 | 0.97 | 0.79 | 0.87 | 1 | 0.99 |
| Phosphoprotein (Tumor Supressor) p53 | 0.6 | 0.96 | 0.98 | 0.9 | 0.86 | 0.89 | 1 | 0.99 |
| ATPase family AAA domain-containing protein 5 (ATAD5) | 0.94 | 0.98 | 0.99 | 0.99 | 0.98 | 0.9 | 1 | 0.99 |


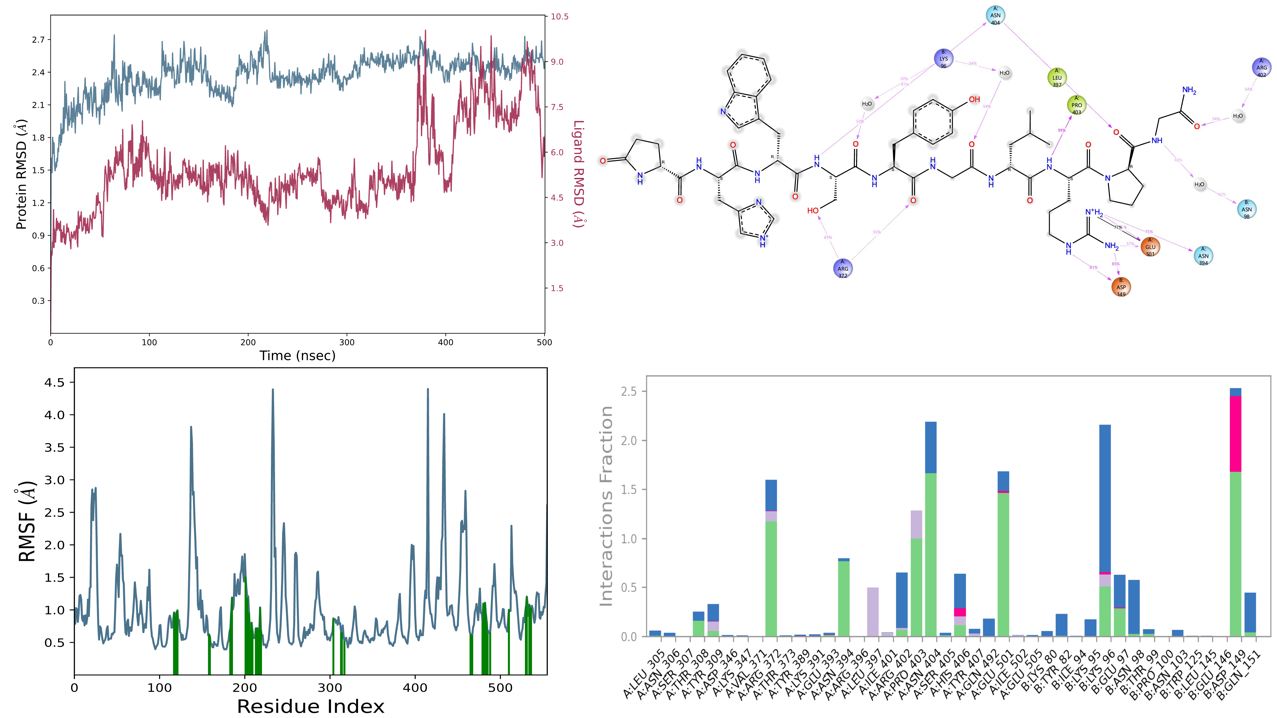


**Figure S5**. Protein and ligand RMSD; 2D interaction diagram; Protein-ligand contacts histogram of the interaction fraction of H-bond (green), hydrophobic bond (Purple), ionic bond (magenta), and water bridges (blue); protein and ligand RMSF of NiV-G-ephrin-B2 structures in complex with DB00644 based on 500 ns MD simulation.


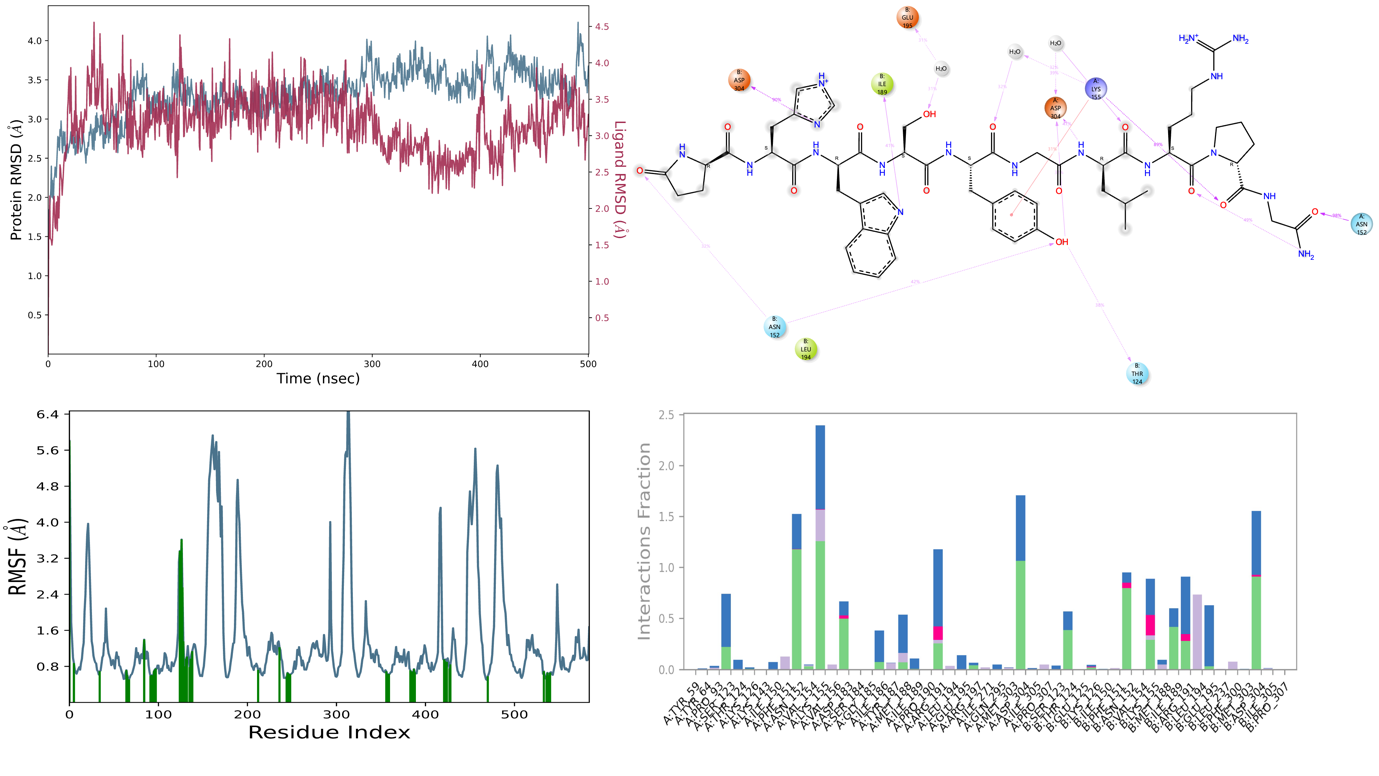


**Figure S6**. Protein and ligand RMSD; 2D interaction diagram; Protein-ligand contacts histogram of the interaction fraction of H-bond (green), hydrophobic bond (Purple), ionic bond (magenta), and water bridges (blue); protein and ligand RMSF of NiV-Matrix protein in complex with DB00644 based on 500 ns MD simulation.


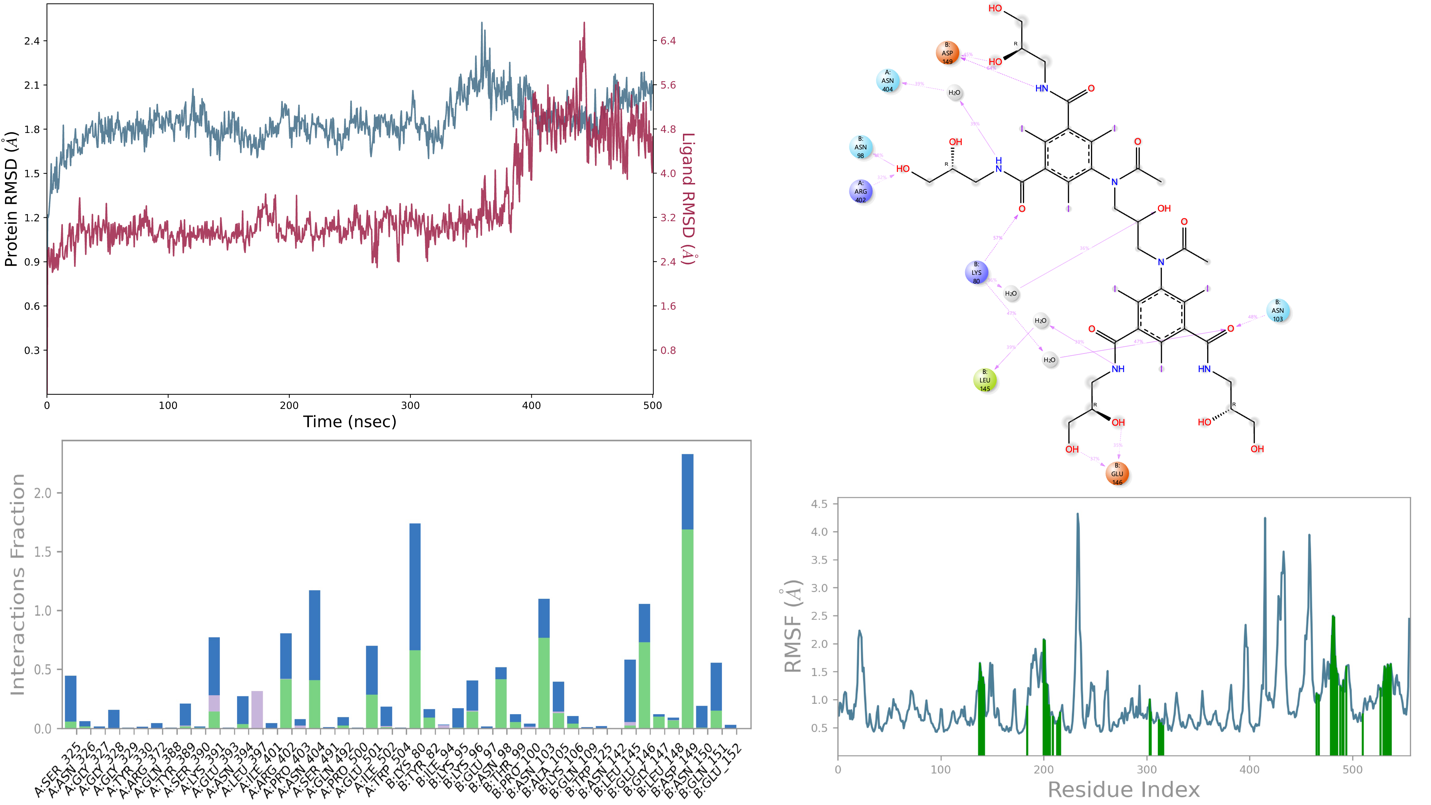


**Figure S7**. Protein and ligand RMSD; 2D interaction diagram; Protein-ligand contacts histogram of the interaction fraction of H-bond (green), hydrophobic bond (Purple), ionic bond (magenta), and water bridges (blue); protein and ligand RMSF of NiV-G-ephrin-B2 structures in complex with DB01249 based on 500 ns MD simulation.


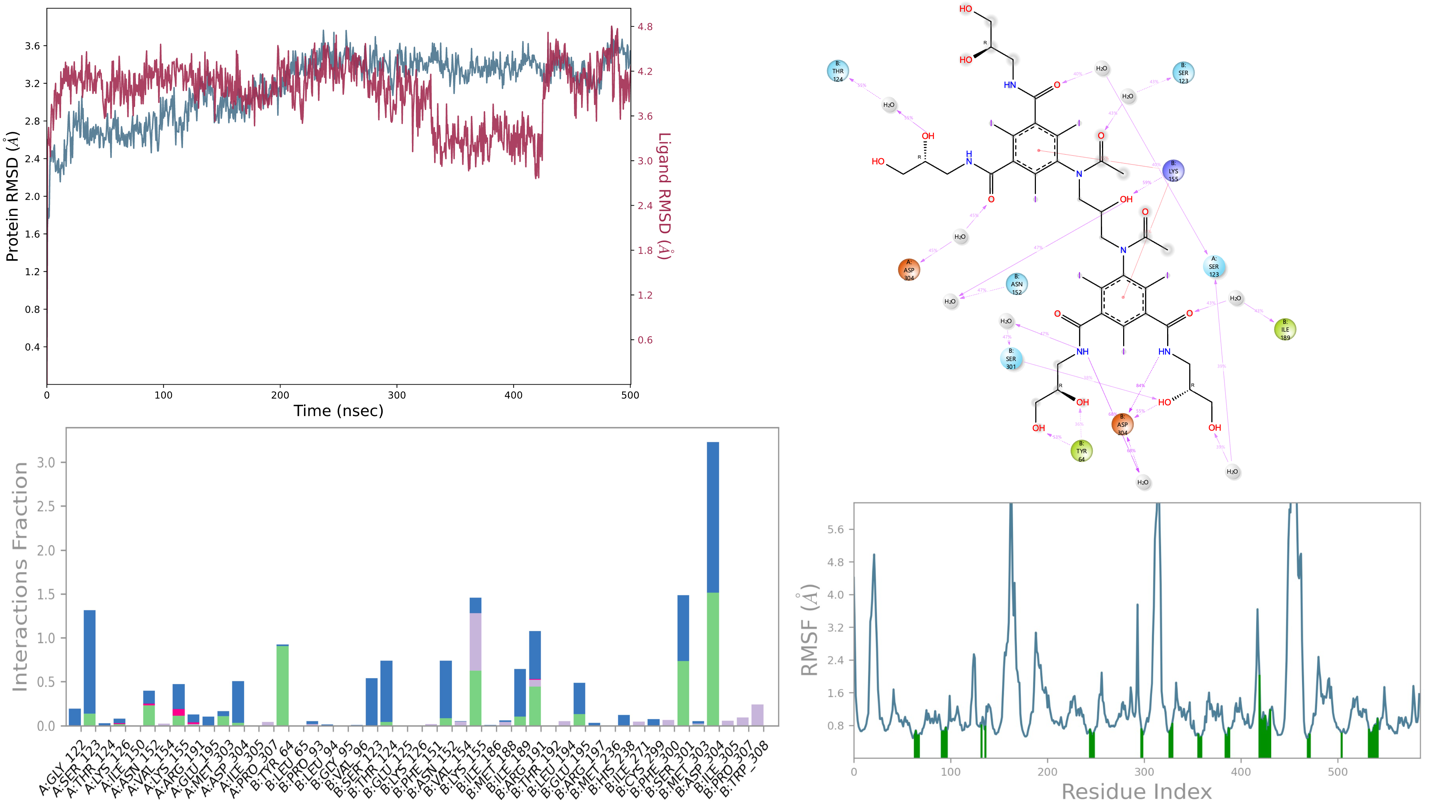


**Figure S8.** Protein and ligand RMSD; 2D interaction diagram; Protein-ligand contacts histogram of the interaction fraction of H-bond (green), hydrophobic bond (Purple), ionic bond (magenta), and water bridges (blue); protein and ligand RMSF of NiV-Matrix protein in complex with DB00644 based on 500 ns MD simulation.


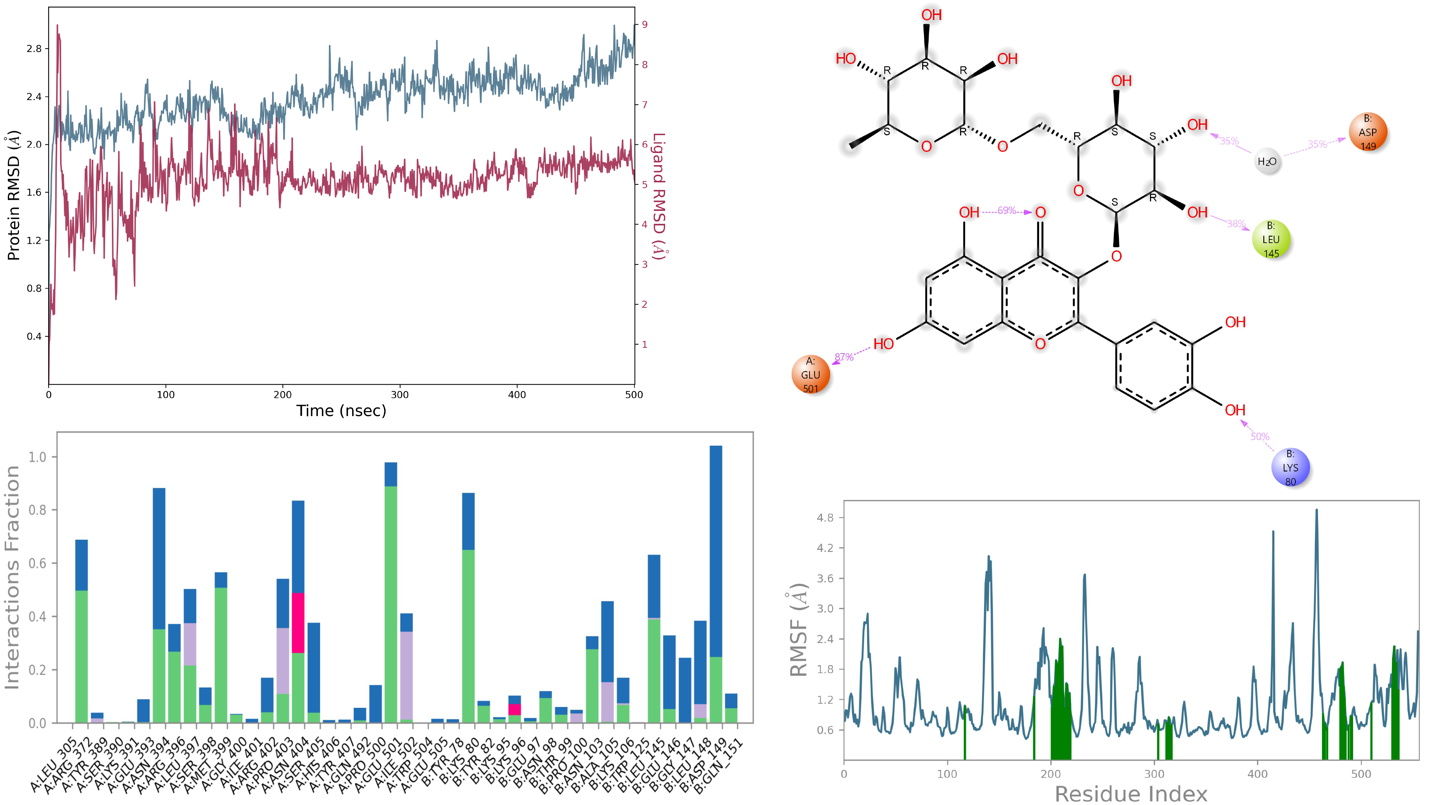


**Figure S9**. Protein and ligand RMSD; 2D interaction diagram; Protein-ligand contacts histogram of the interaction fraction of H-bond (green), hydrophobic bond (Purple), ionic bond (magenta), and water bridges (blue); protein and ligand RMSF of NiV-G-ephrin-B2 structures in complex with DB01698 based on 500 ns MD simulation.


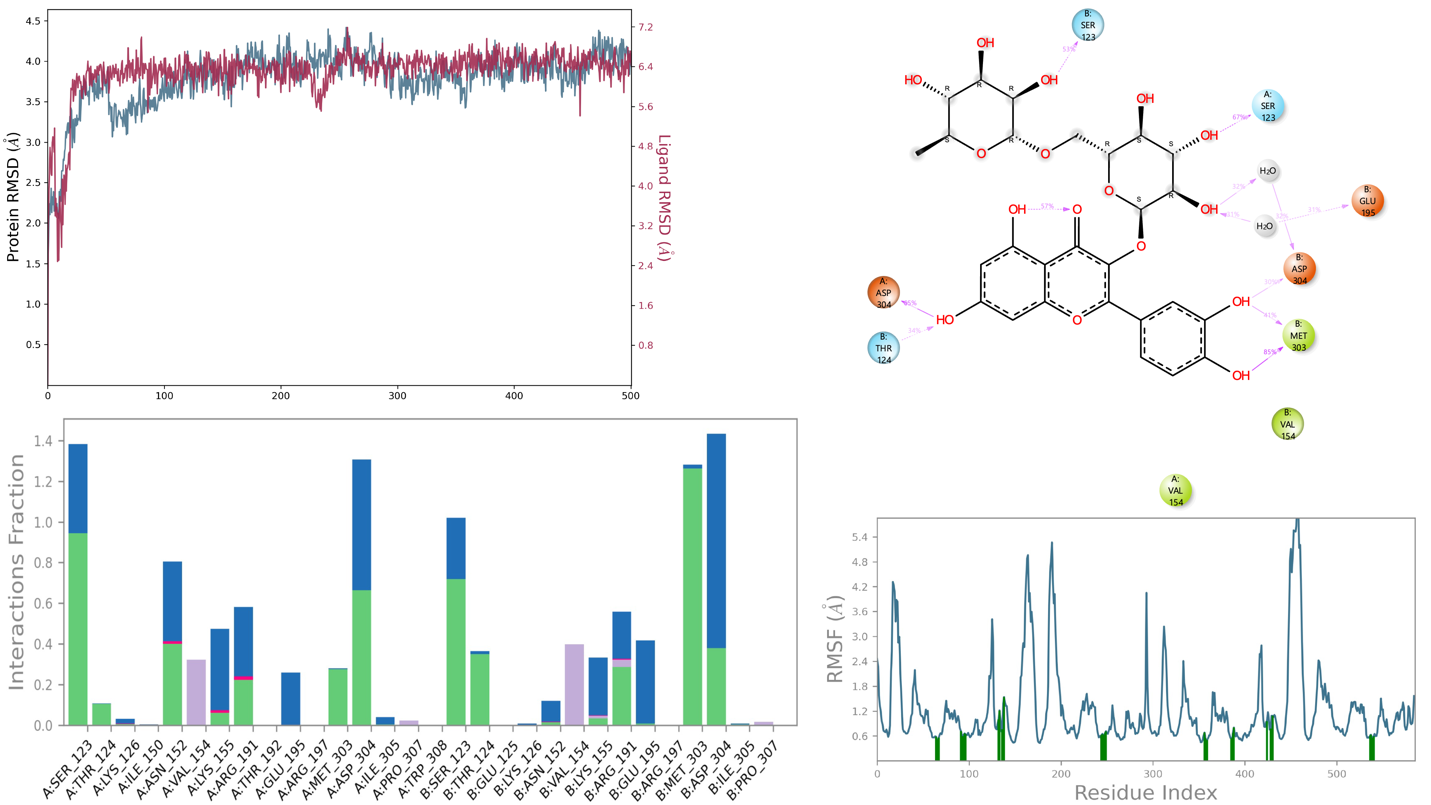


**Figure S10**. Protein and ligand RMSD; 2D interaction diagram; Protein-ligand contacts histogram of the interaction fraction of H-bond (green), hydrophobic bond (Purple), ionic bond (magenta), and water bridges (blue); protein and ligand RMSF of NiV-Matrix protein in complex with DB01698 based on 500 ns MD simulation.


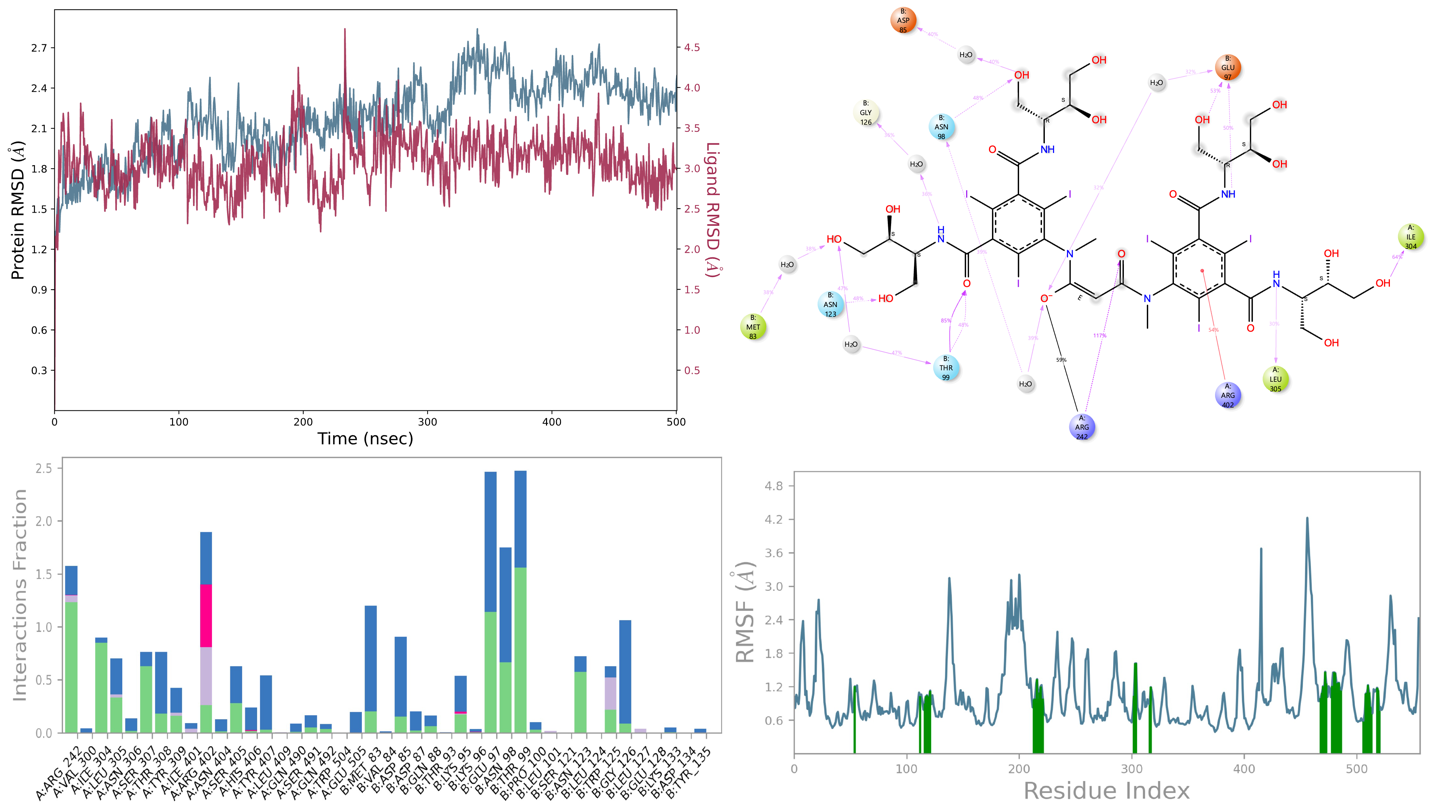


**Figure S11**. Protein and ligand RMSD; 2D interaction diagram; Protein-ligand contacts histogram of the interaction fraction of H-bond (green), hydrophobic bond (Purple), ionic bond (magenta), and water bridges (blue); protein and ligand RMSF of NiV-G-ephrin-B2 structures in complex with DB09487 based on 500 ns MD simulation.


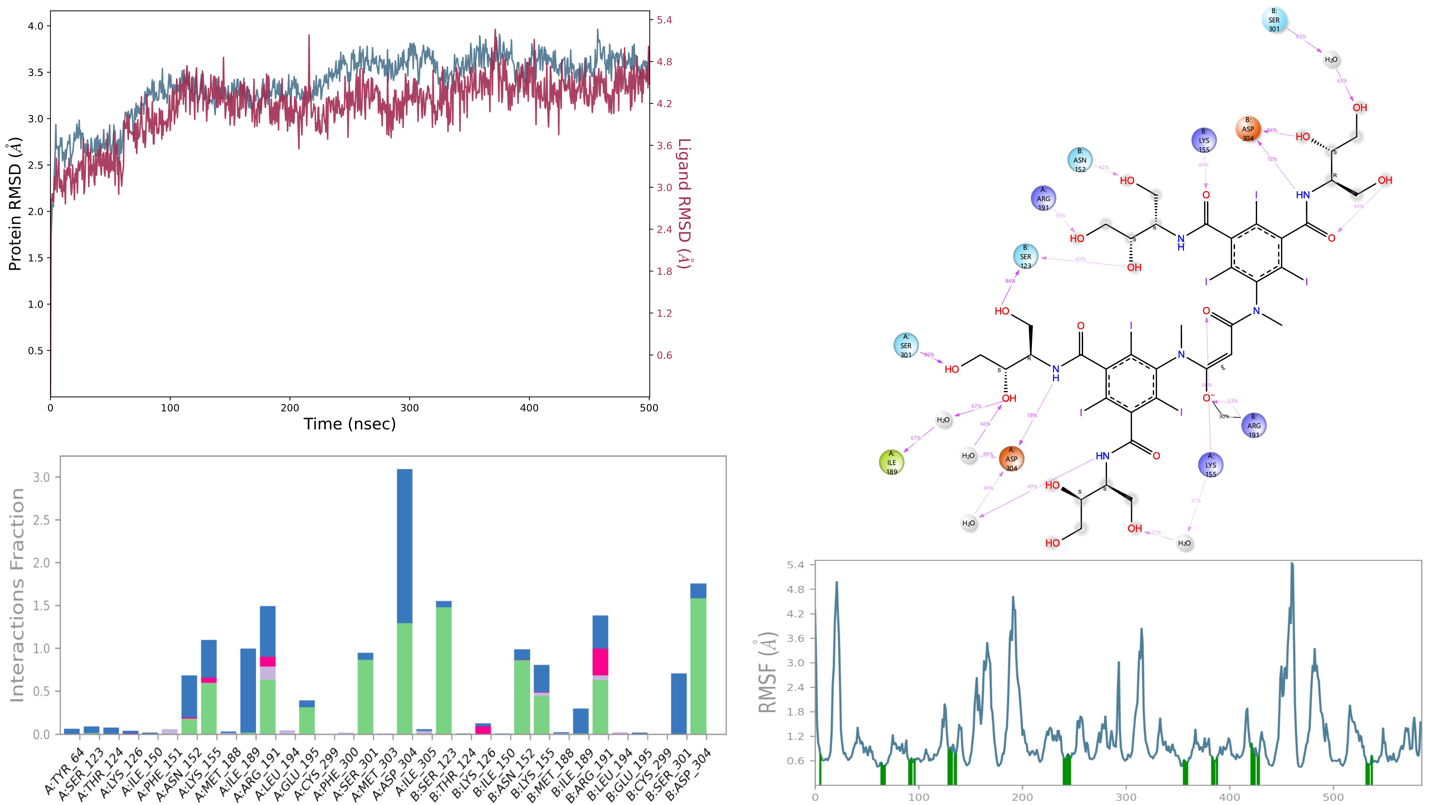


**Figure S12**. Protein and ligand RMSD; 2D interaction diagram; Protein-ligand contacts histogram of the interaction fraction of H-bond (green), hydrophobic bond (Purple), ionic bond (magenta), and water bridges (blue); protein and ligand RMSF of NiV-Matrix protein in complex with DB09487 based on 500 ns MD simulation.


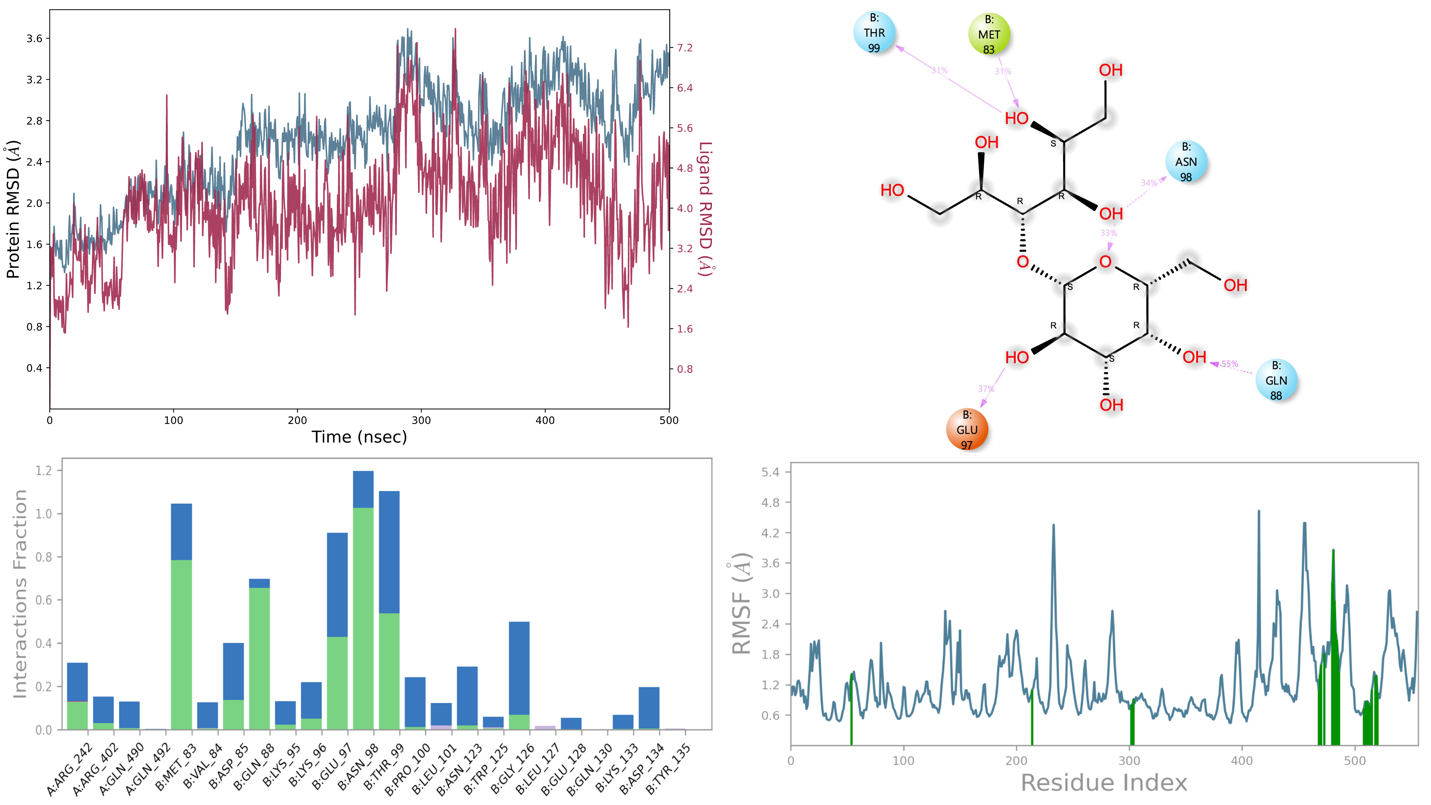


**Figure S13**. Protein and ligand RMSD; 2D interaction diagram; Protein-ligand contacts histogram of the interaction fraction of H-bond (green), hydrophobic bond (Purple), ionic bond (magenta), and water bridges (blue); protein and ligand RMSF of NiV-G-ephrin-B2 structures in complex with DB12942 based on 500 ns MD simulation.


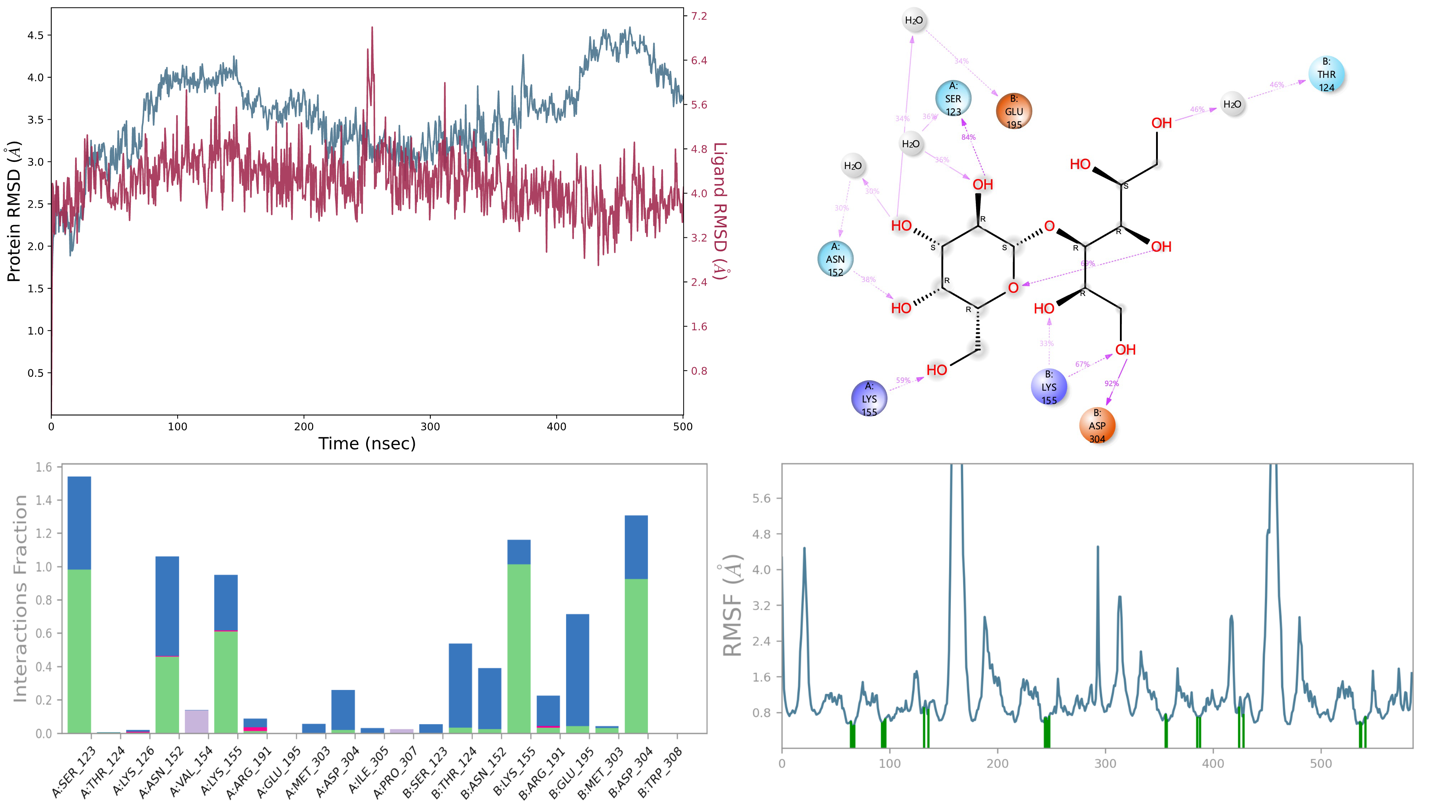


**Figure S14**. Protein and ligand RMSD; 2D interaction diagram; Protein-ligand contacts histogram of the interaction fraction of H-bond (green), hydrophobic bond (Purple), ionic bond (magenta), and water bridges (blue); protein and ligand RMSF of NiV-Matrix protein in complex with DB12942 based on 500 ns MD simulation.


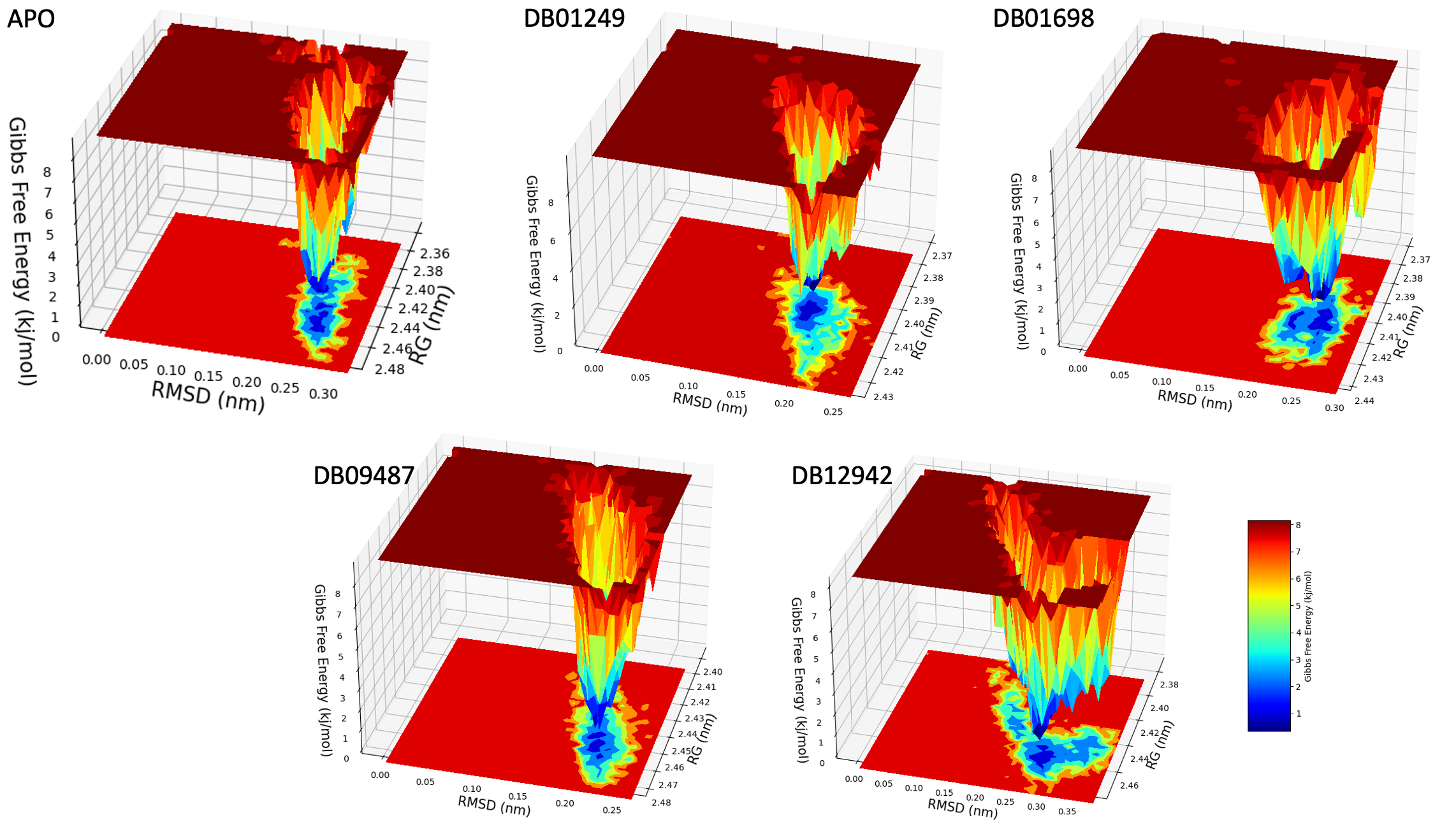


**Figure S15**. The free energy landscape (FEL) of 2VSM protein with and without ligands with color-coded scale.


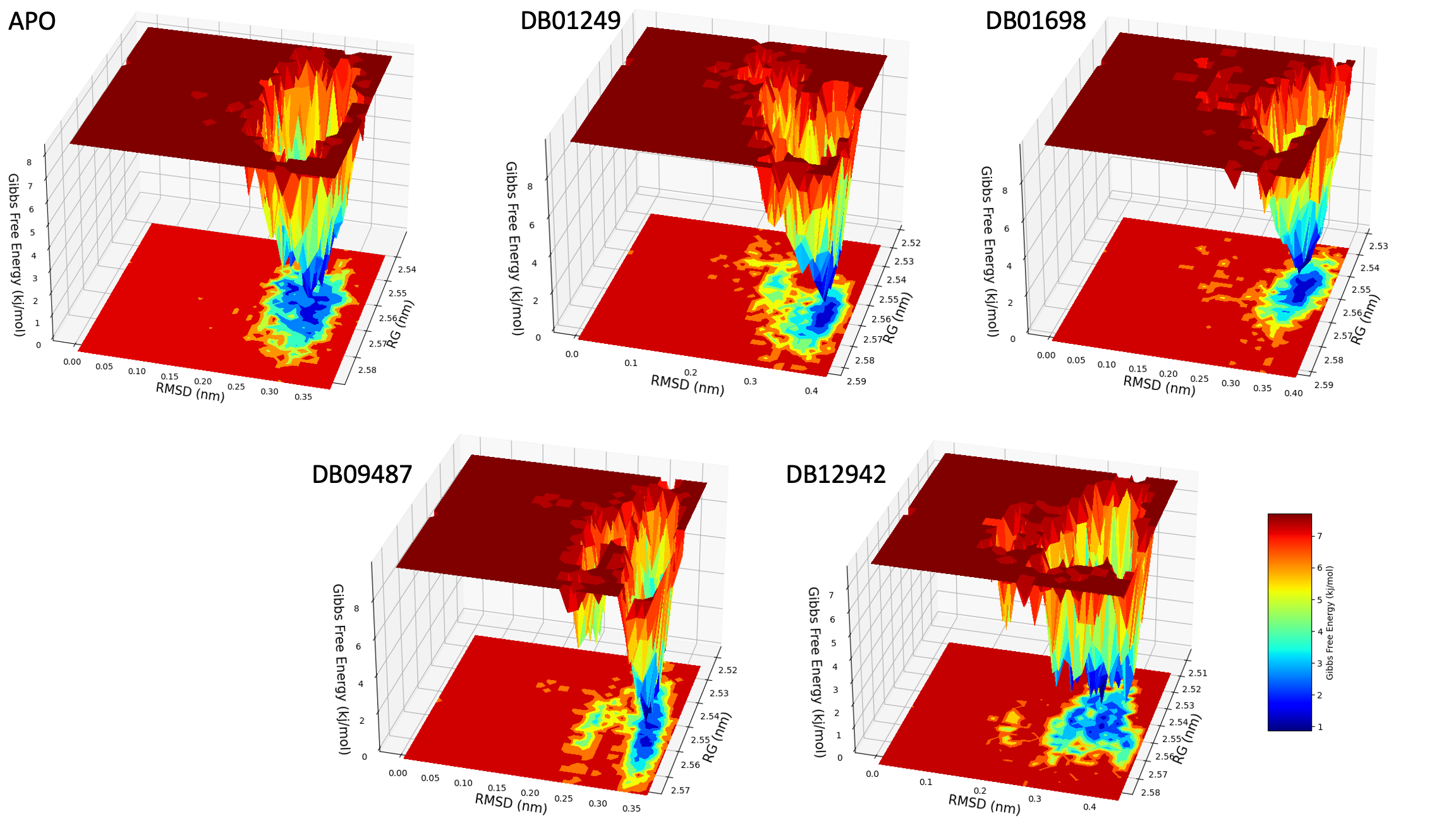


**Figure S16**. The free energy landscape (FEL) of 7SKT protein with and without ligands with color-coded scale.


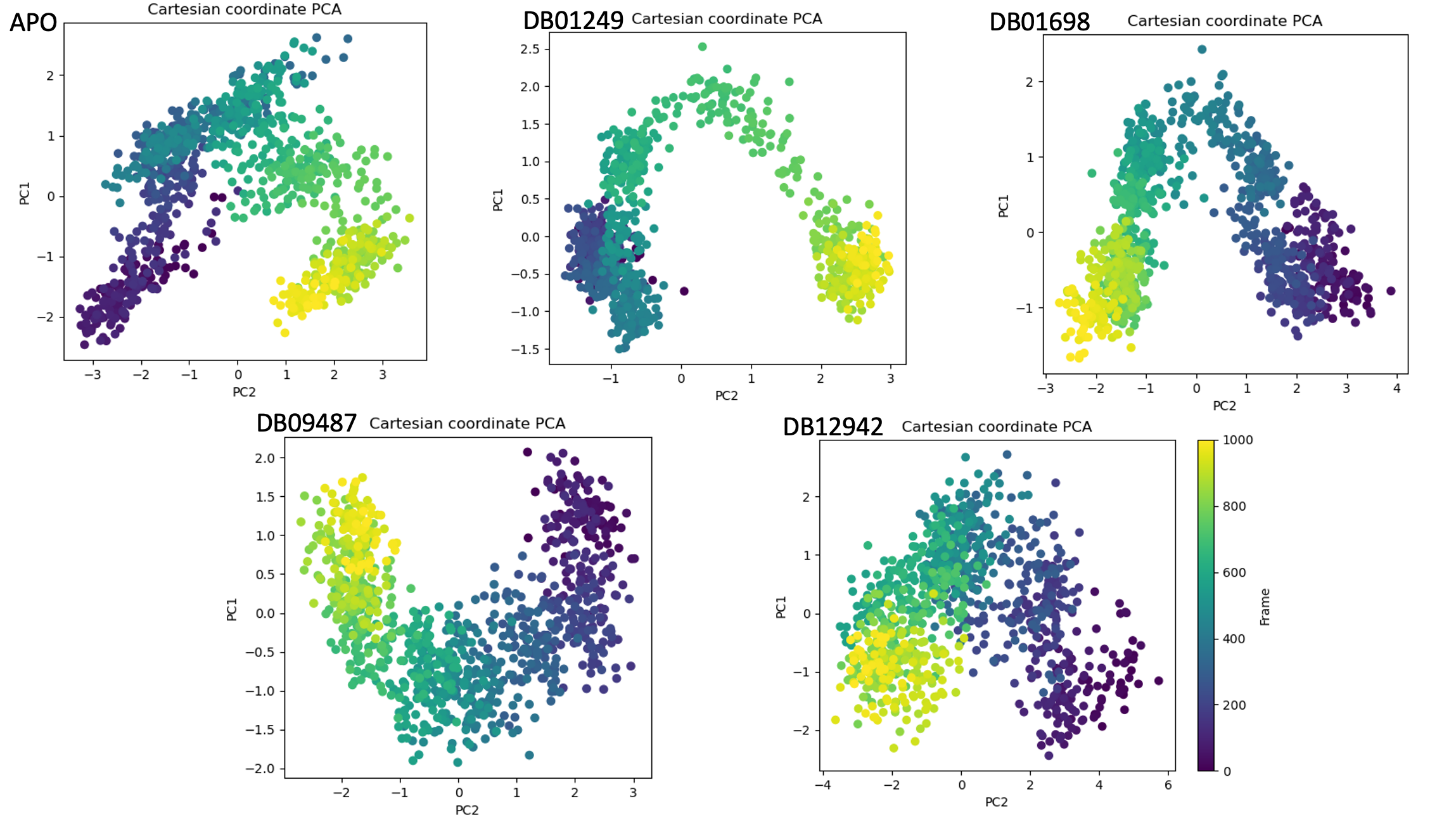


**Figure S17**. Principle component analysis (PCA) of 2VSM over time in Apo and ligand-bound state.


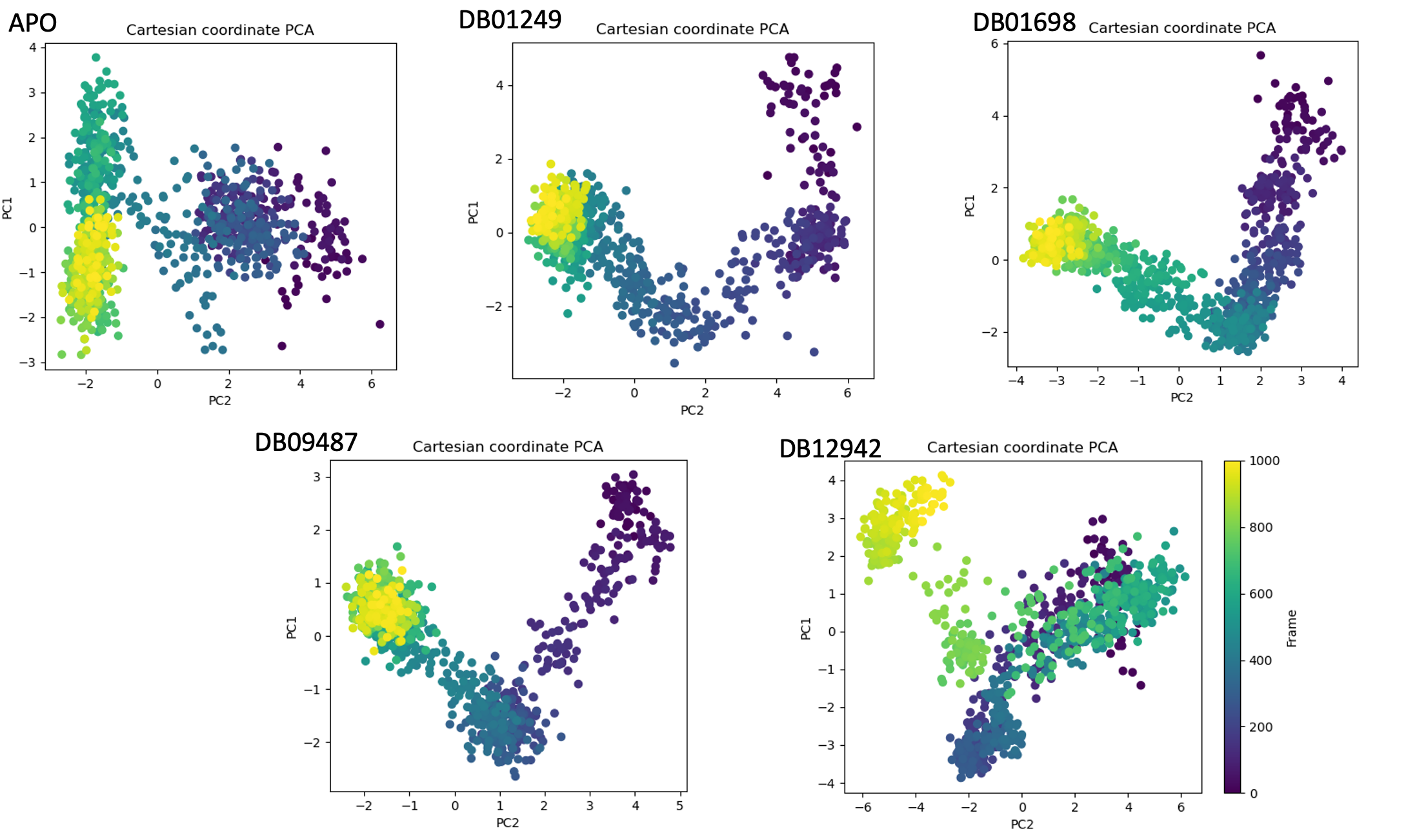


**Figure S18.** Principle component analysis (PCA) of 7SKT over time in Apo and ligand-bound state.


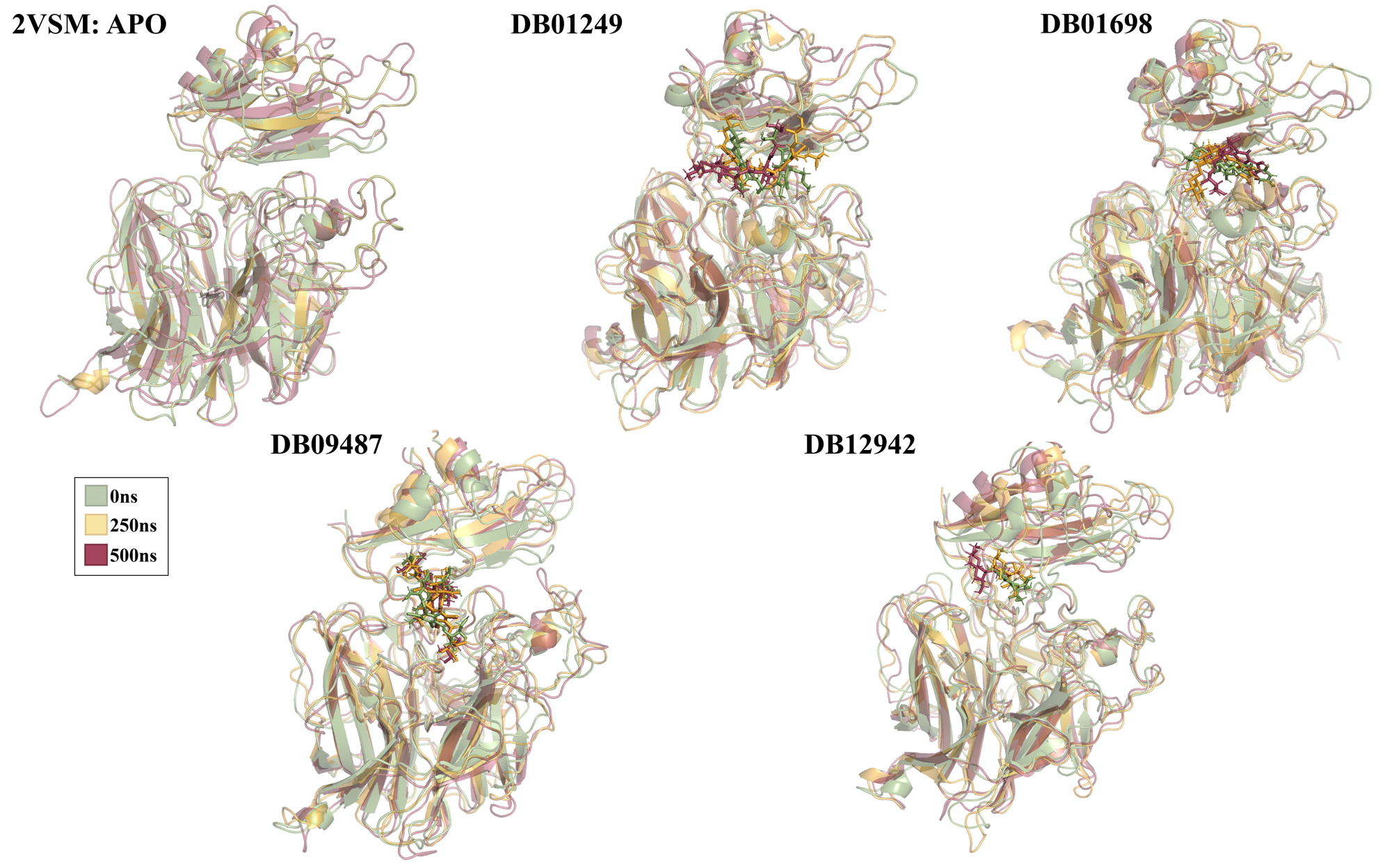


**Figure S19**. Comparative secondary structure evolution of NiV-G protein (2VSM) over time in Apo and ligand-bound states at three simulation time points: 0 ns (green), 250 ns (orange), and 500 ns (red).


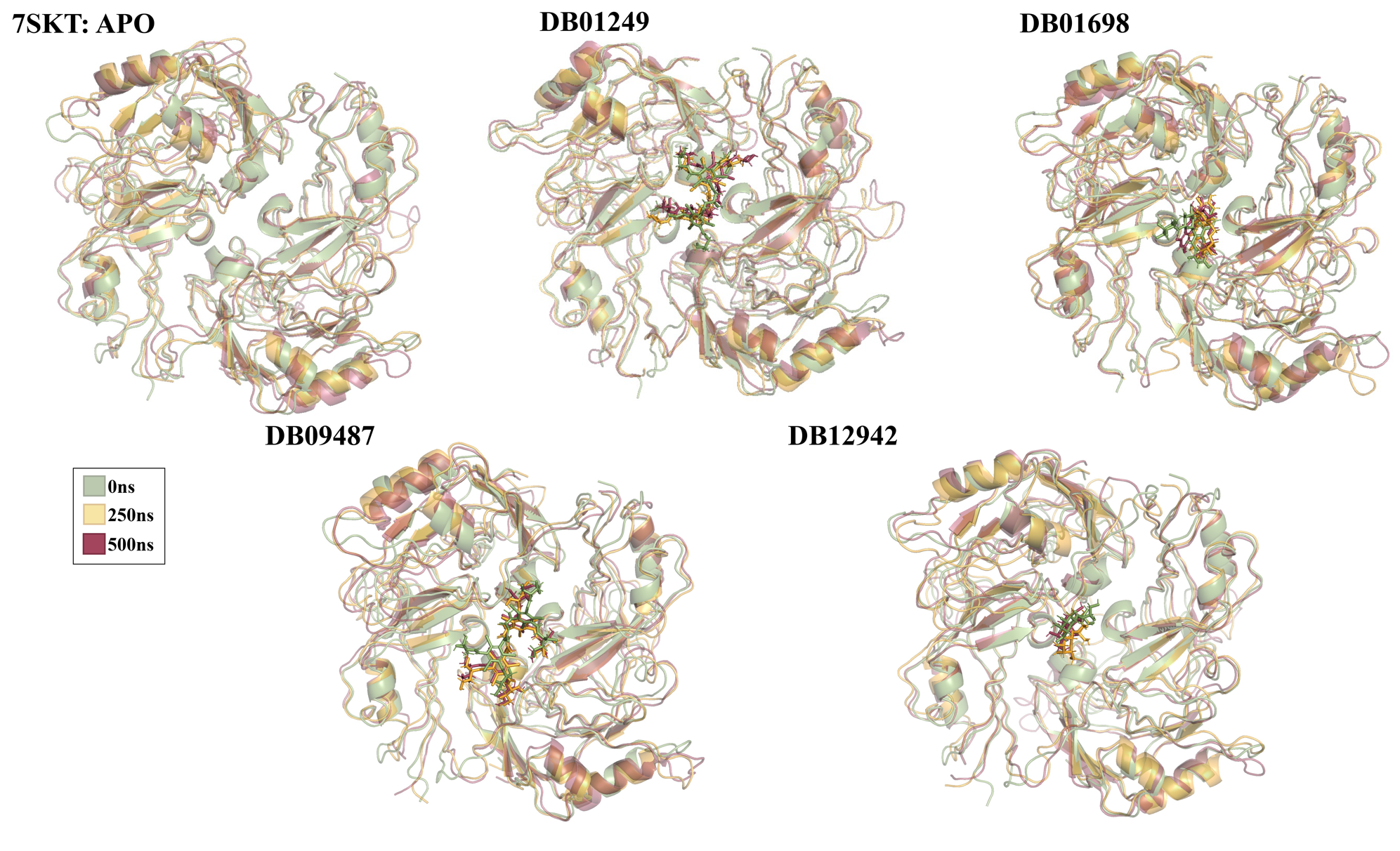


**Figure S20**. Comparative secondary structure evolution of NiV-M protein (7SKT) over time in Apo and ligand-bound states at three simulation time points: 0 ns (green), 250 ns (orange), and 500 ns (red).


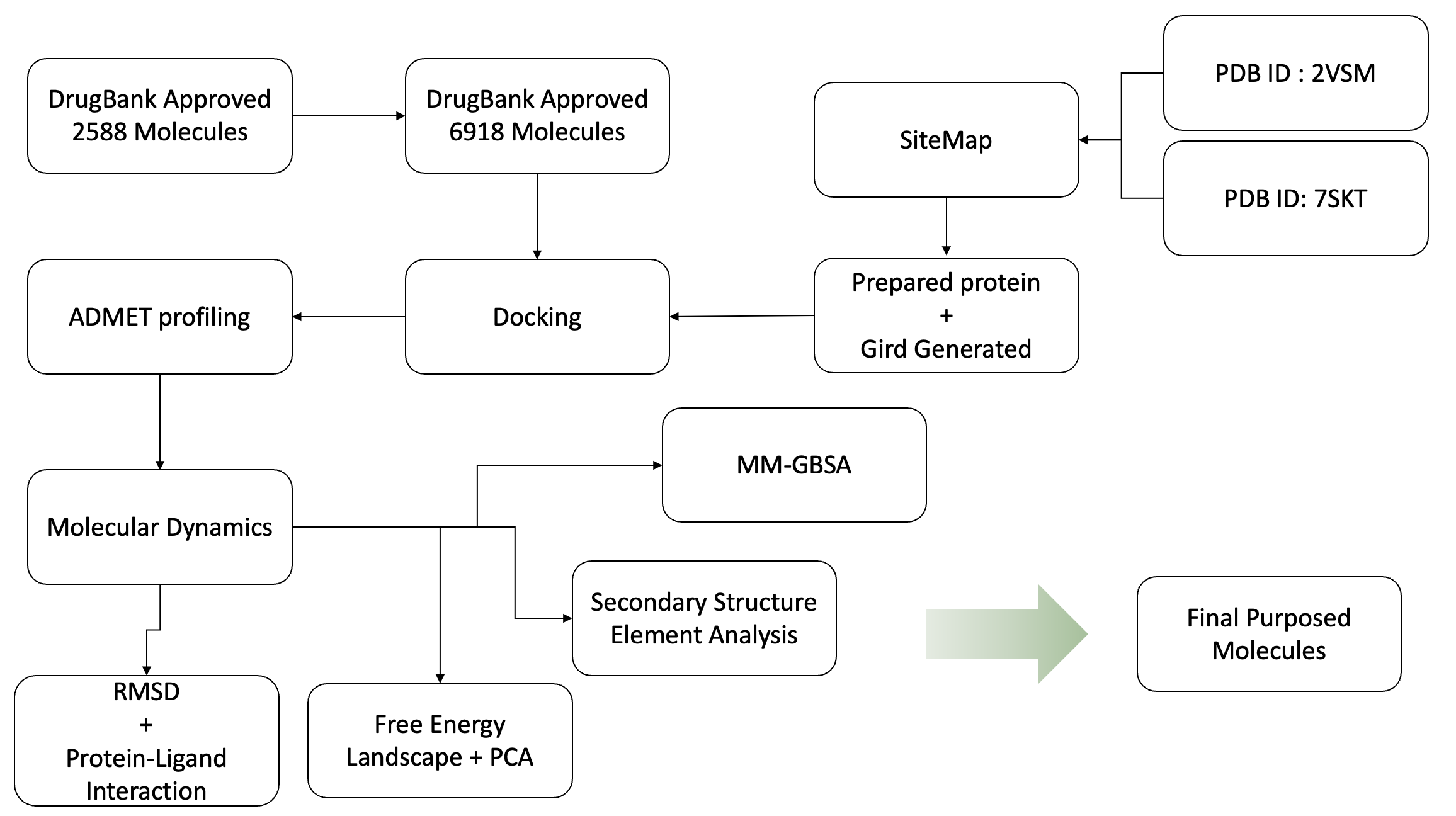


**Figure S21**. Workflow diagram for the inhibitor identification process.
